# Supplementary material for: Playbook workflow builder: Interactive construction of bioinformatics workflows
Source: PLoS Comput Biol. 2025 Apr 3;21(4):e1012901. doi: 10.1371/journal.pcbi.1012901 (PMC11967941; doi:10.1371/journal.pcbi.1012901)

Fig. S1 A

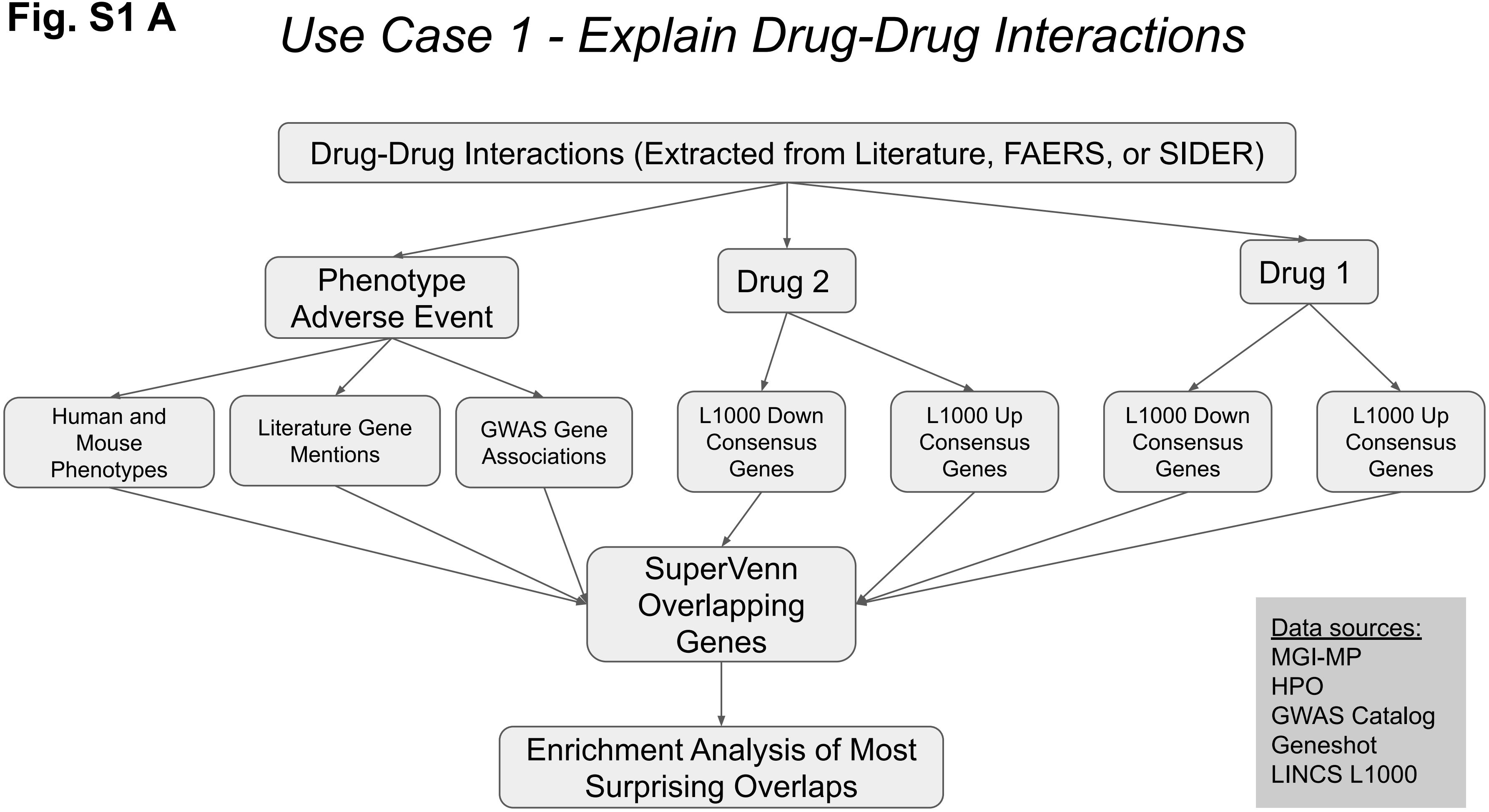

Use Case 1 - Explain Drug-Drug Interactions
Drug-Drug Interactions (Extracted from Literature, FAERS, or SIDER)
Phenotype Adverse Event
Drug 2
Drug 1
Human and Mouse Phenotypes
Literature Gene Mentions
GWAS Gene Associations
L1000 Down Consensus Genes
L1000 Up Consensus Genes
L1000 Down Consensus Genes
L1000 Up Consensus Genes
SuperVenn Overlapping Genes
Enrichment Analysis of Most Surprising Overlaps
Data sources:
MGI-MP
HPO
GWAS Catalog
Geneshot
LINCS L1000

Use Case 2 - Explain MOAs of Side Effects for Approved Drugs

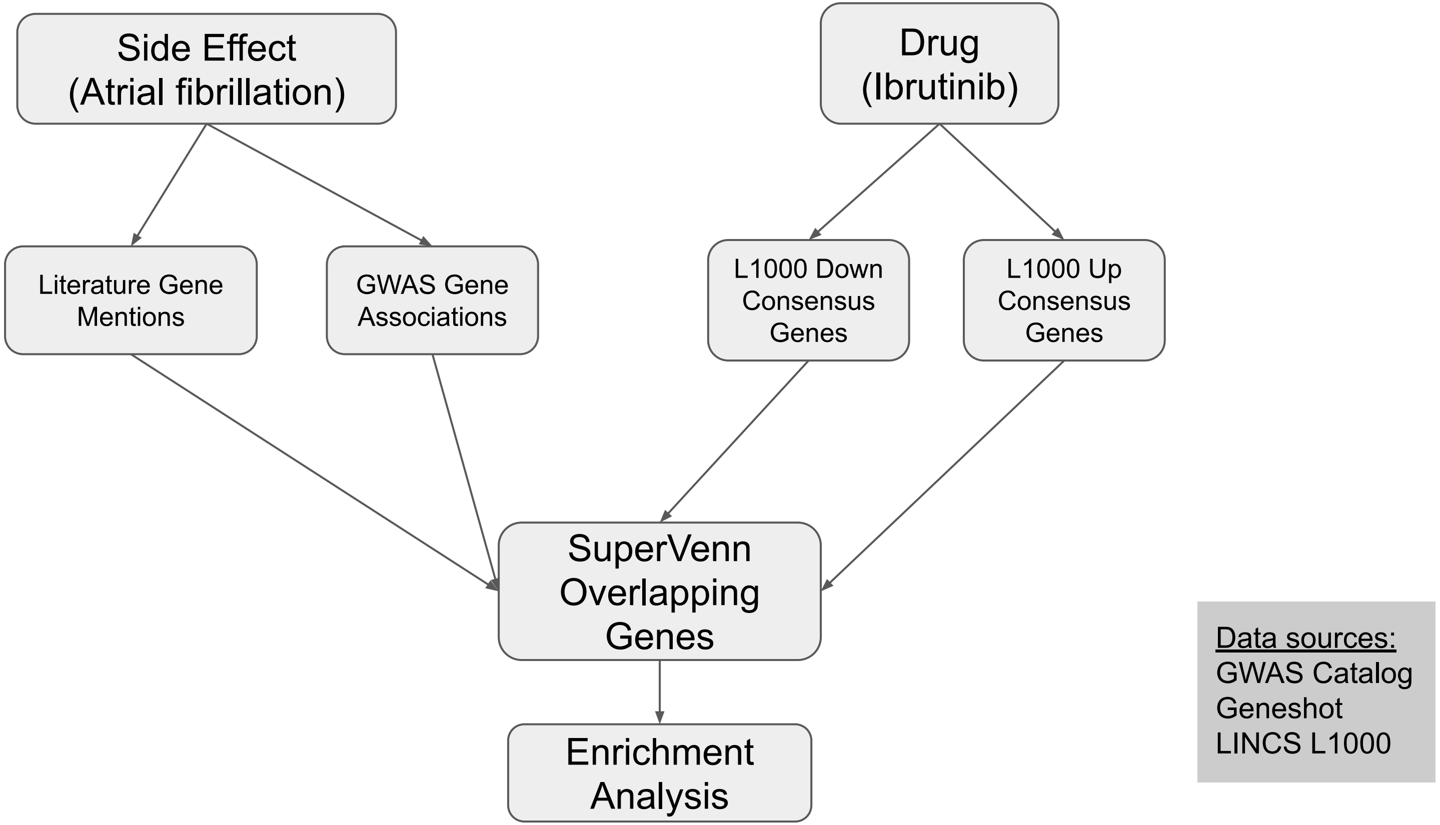

Fig. S1 B

Use Case 3 - Compounds to Reverse Disease Signatures

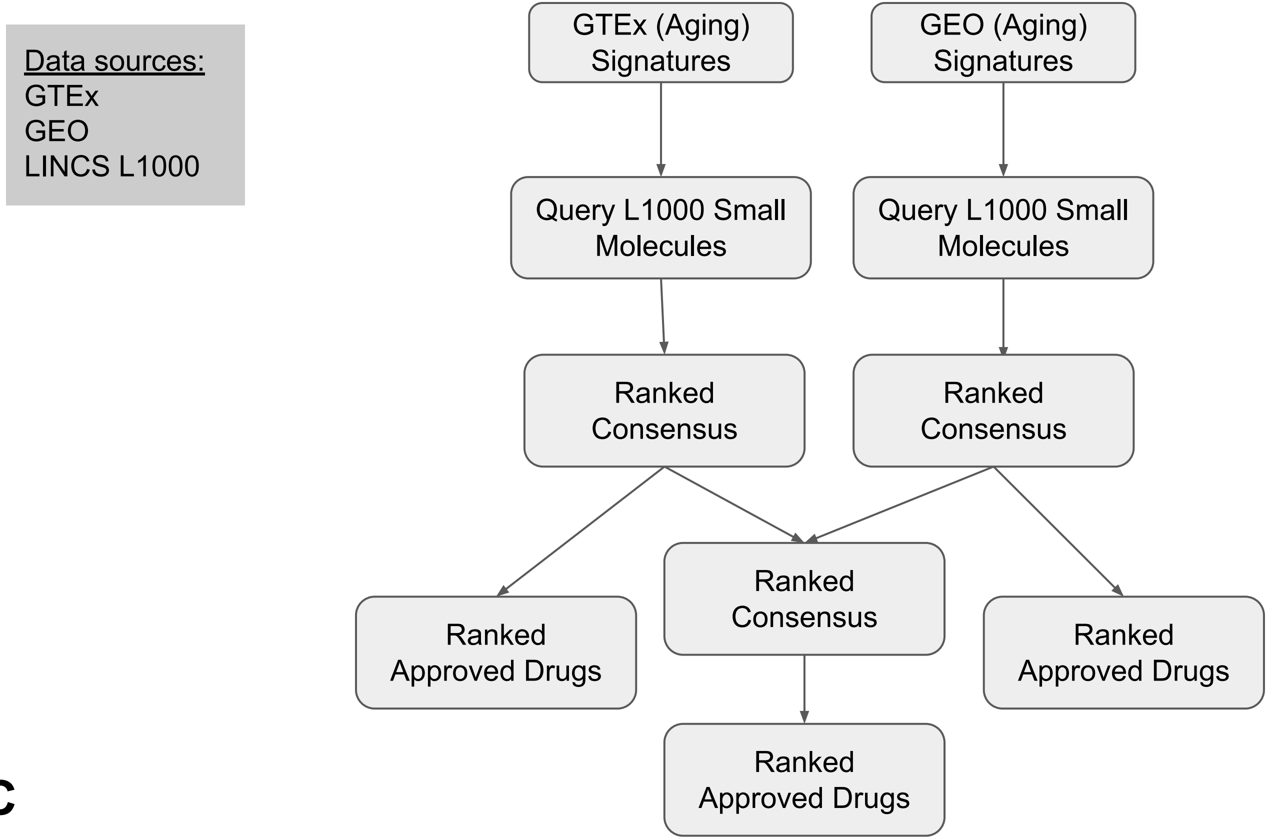

Fig. S1 C

Fig. S1 D

Use Case 4 - KLF4 Targets in GTEx

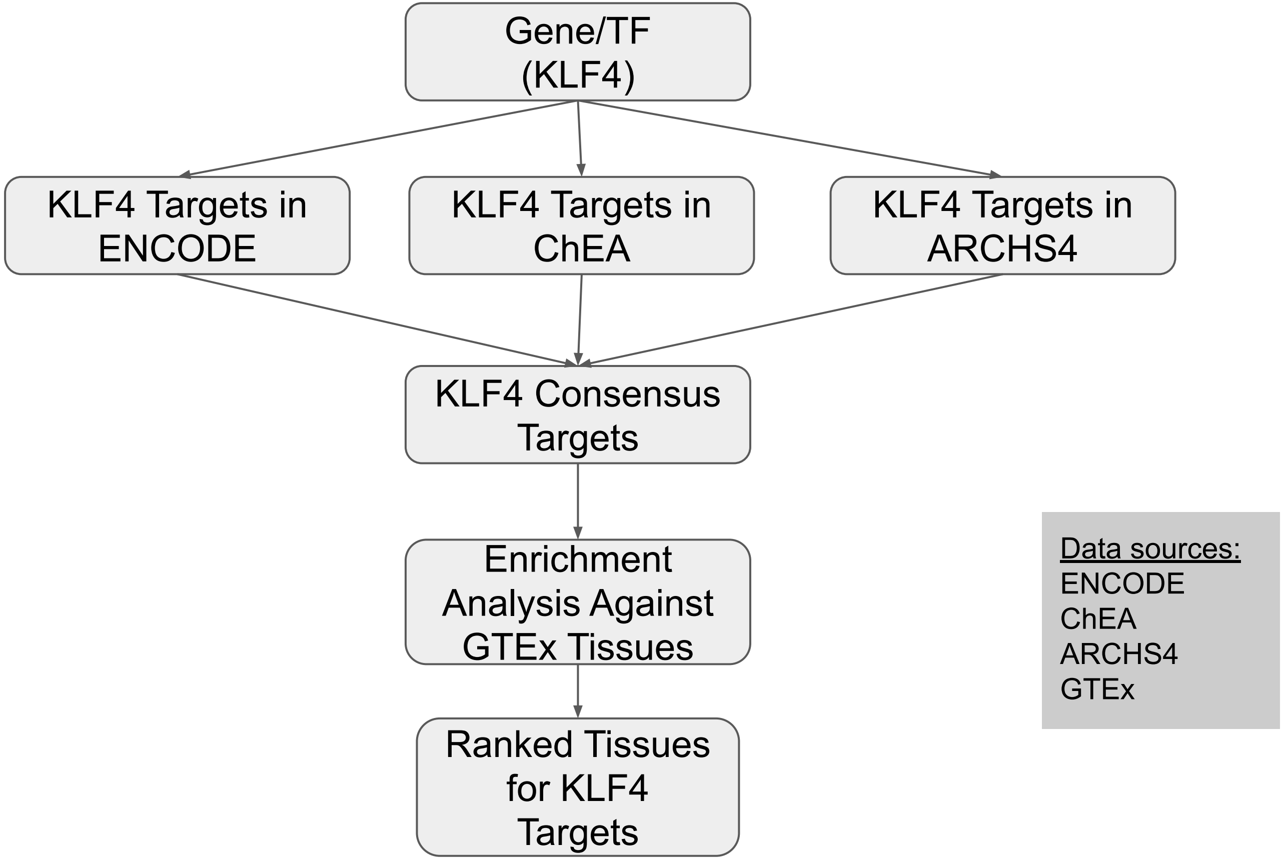

Use Case 5 - Small Molecules to Induce a Biological Process (e.g. Autophagy)

Data sources:
HPO
MGI-MP
KEGG
WikiPathways
GO
LINCS L1000

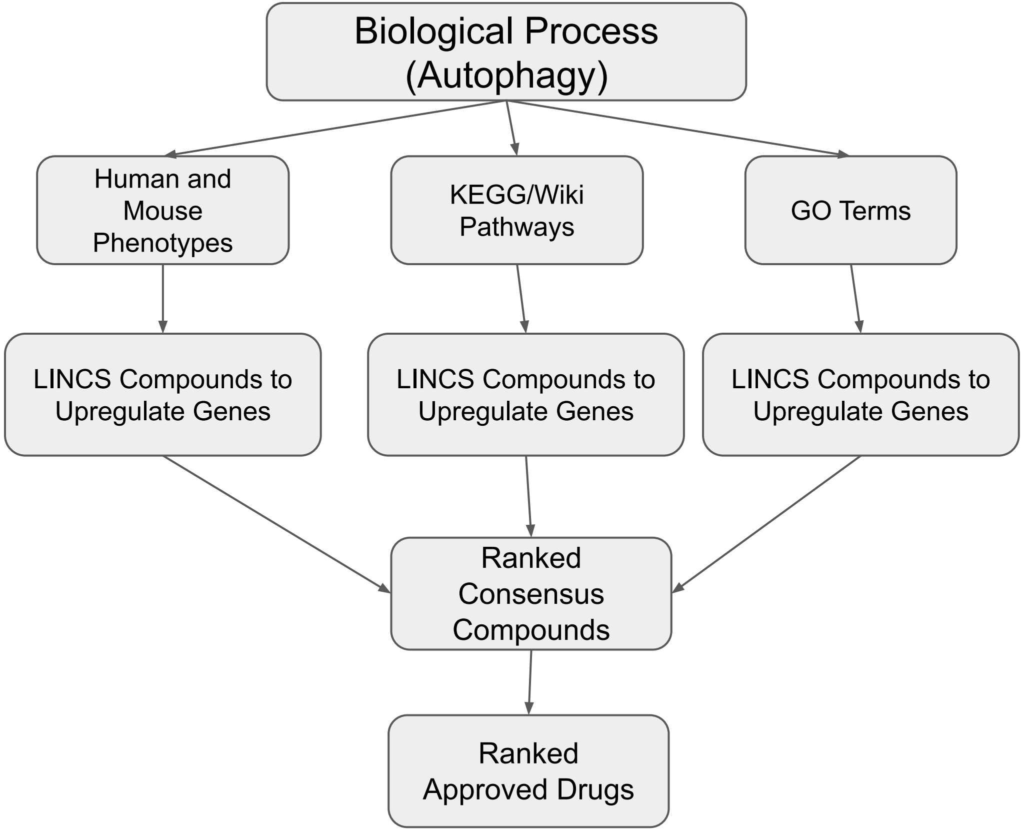

Fig. S1 E

Fig. S1 F *Use Case 6 - CFDE Knowledge about a Gene (KLF6)*

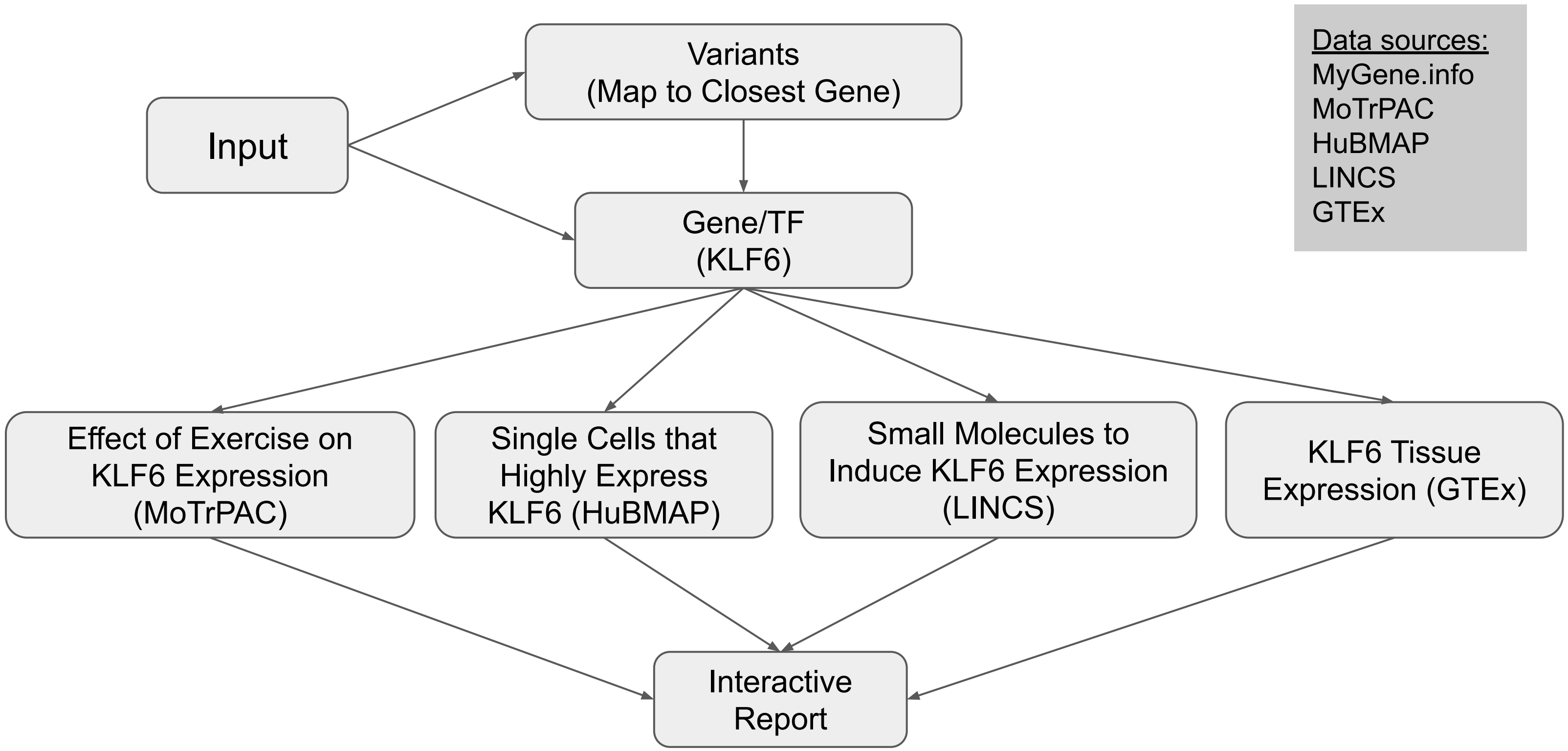

Fig. S1 G

Use Case 7 - Gene/Variant Expression

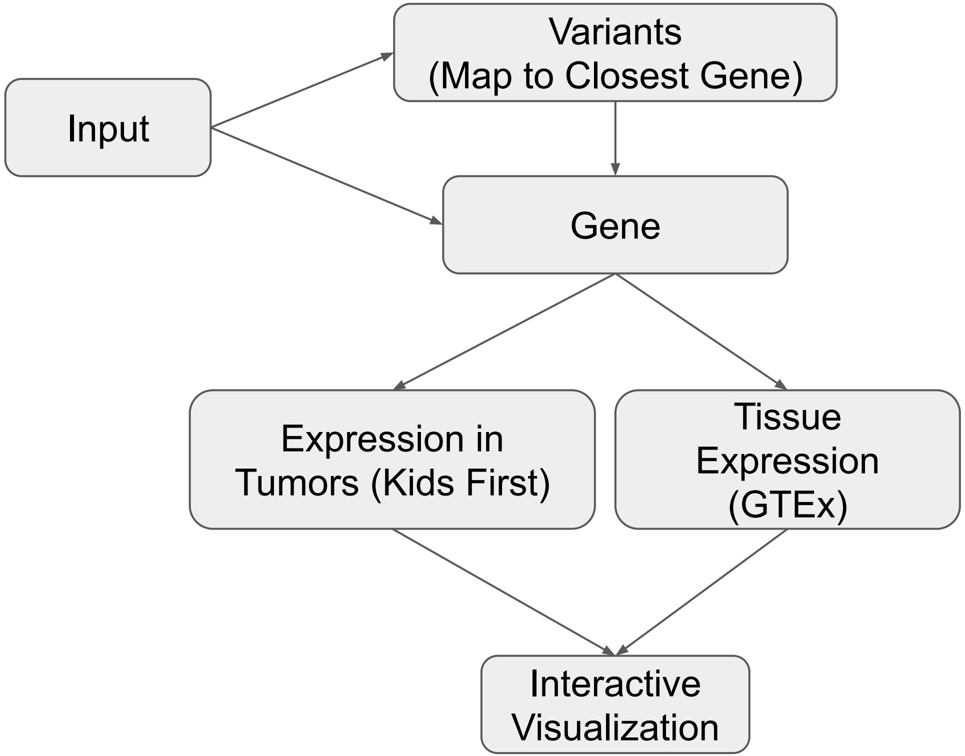

<u>Data sources:</u>
Kids First
GTEx

Use Case 8 - Associations between 2 Genes/Variants

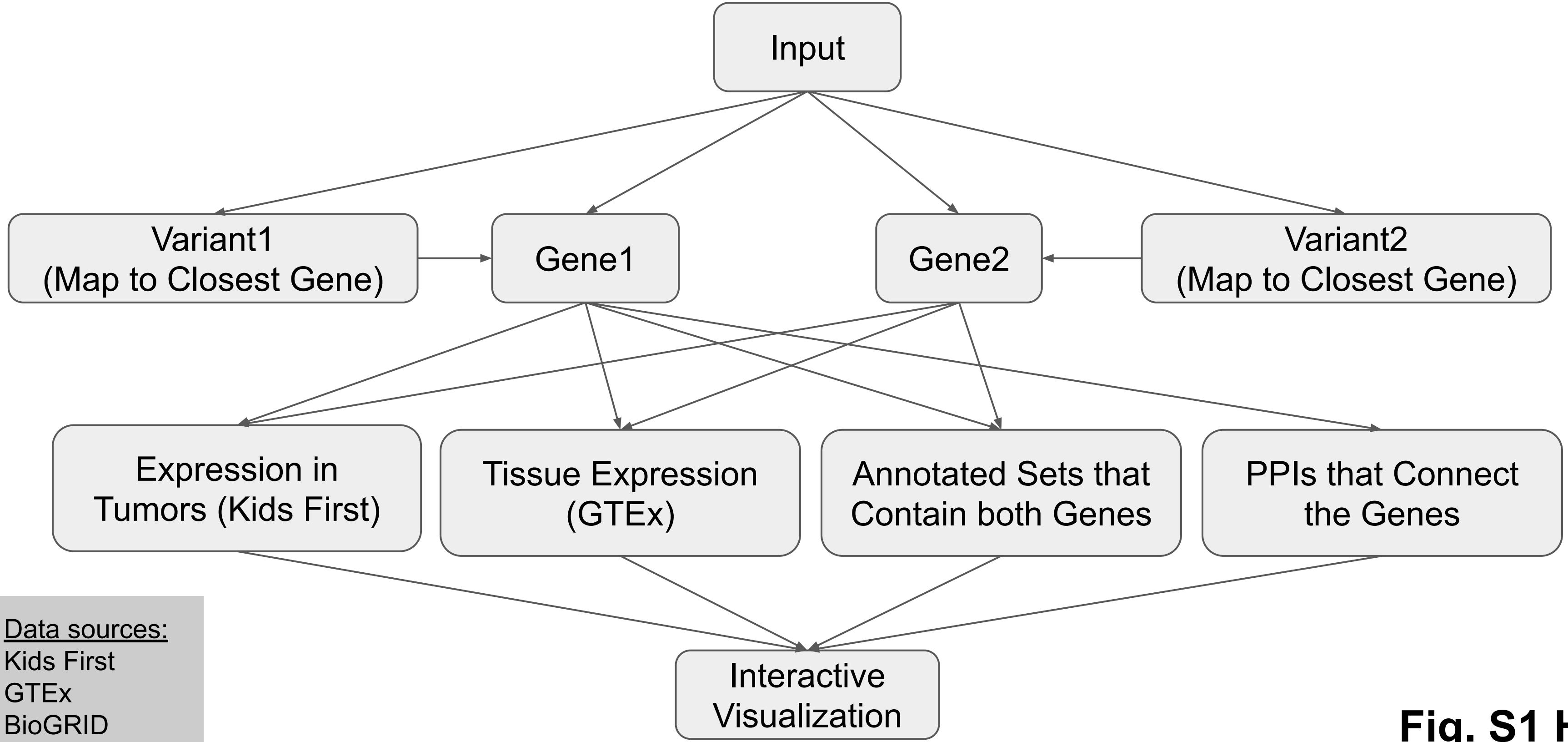

Data sources:
Kids First
GTEx
BioGRID
MyGene.info

Fig. S1 H

Use Case 9 - Identifying Regulatory Relationships between Genes, Regulatory Regions, and Variants using FAIR Information and Knowledge

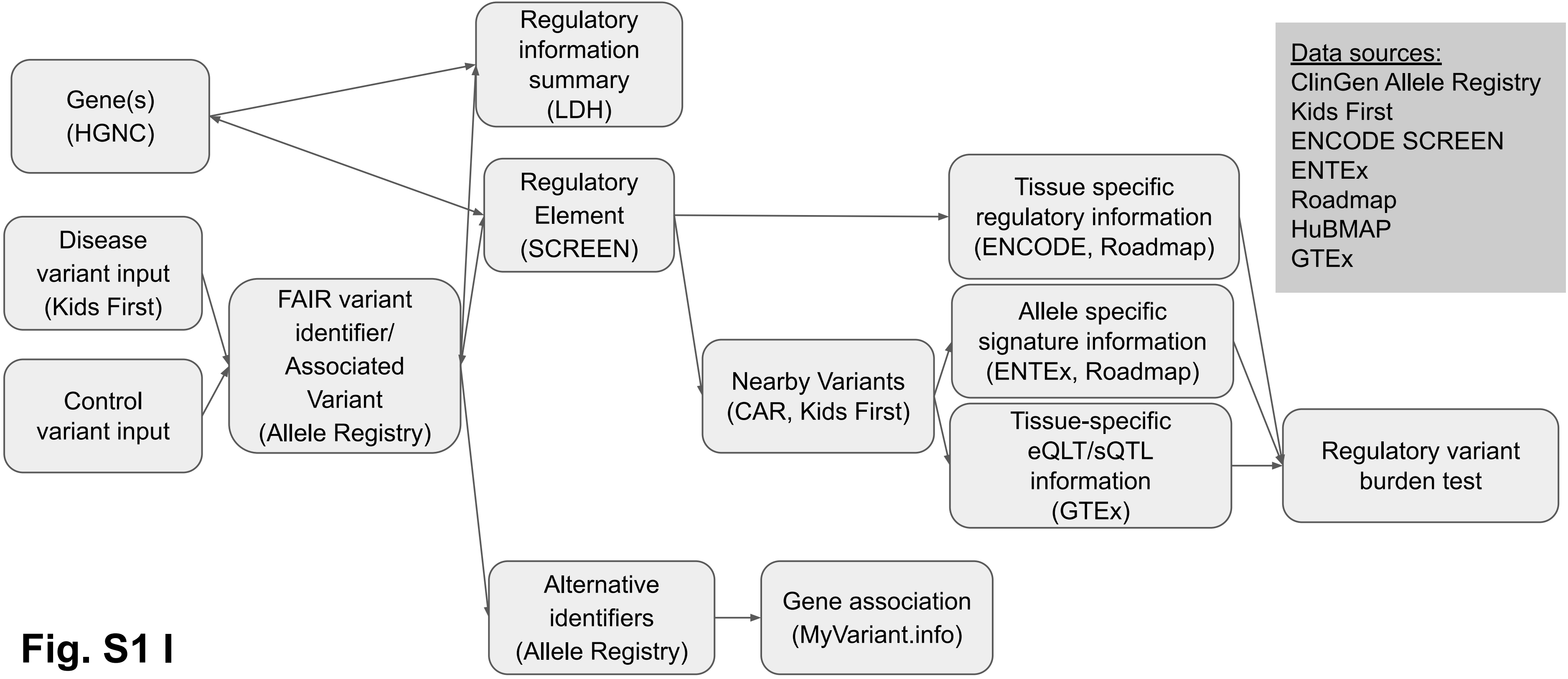

Fig. S1 I

Use Case 12 - SNVs Impact on Glycosylated Sites and Glycogenes Expression

Fig. S1 J

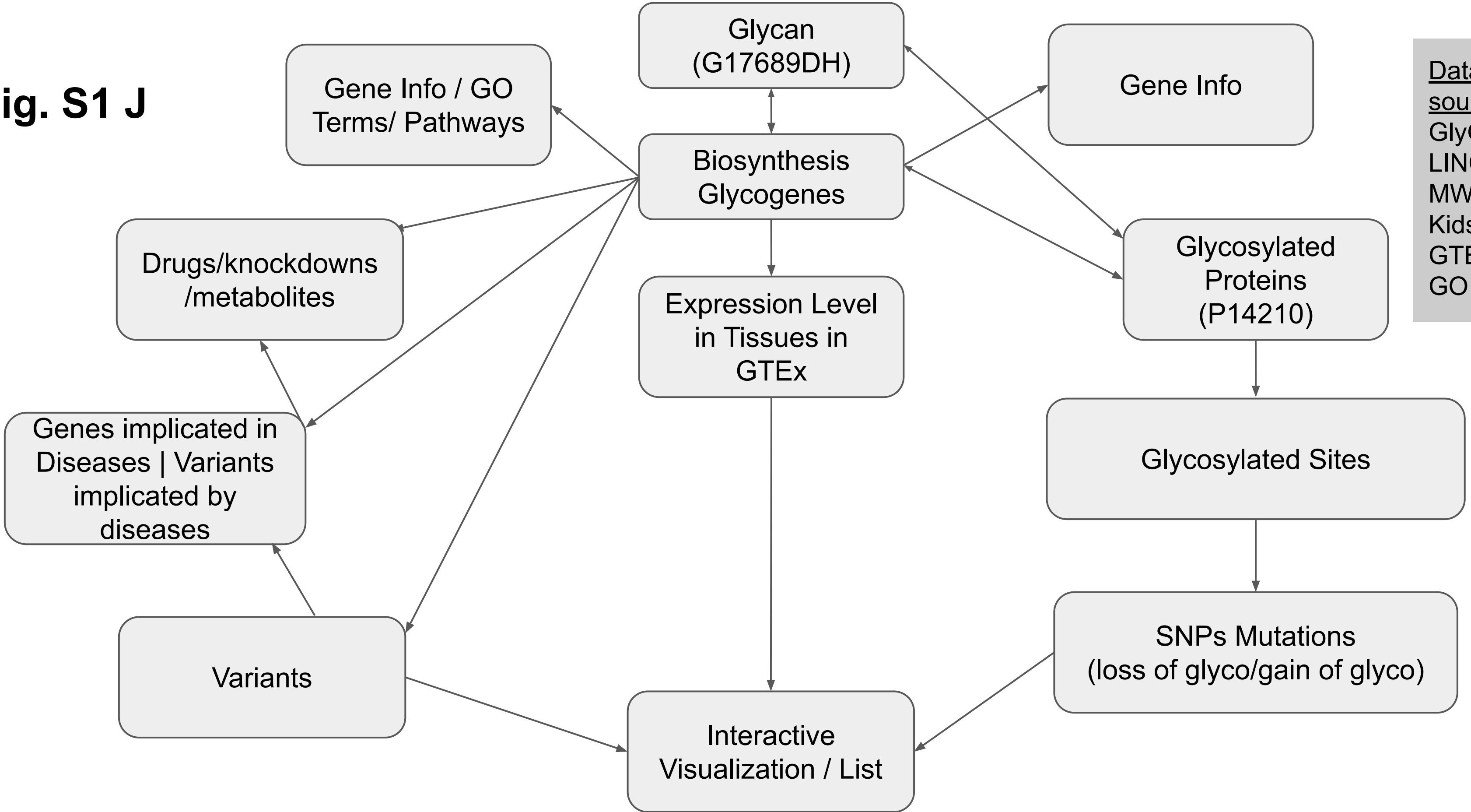

Data sources:
GlyGen
LINCS
MW
Kids First
GTEx
GO

Use Case 13 - Prioritizing Targets for Individual Cancer patients

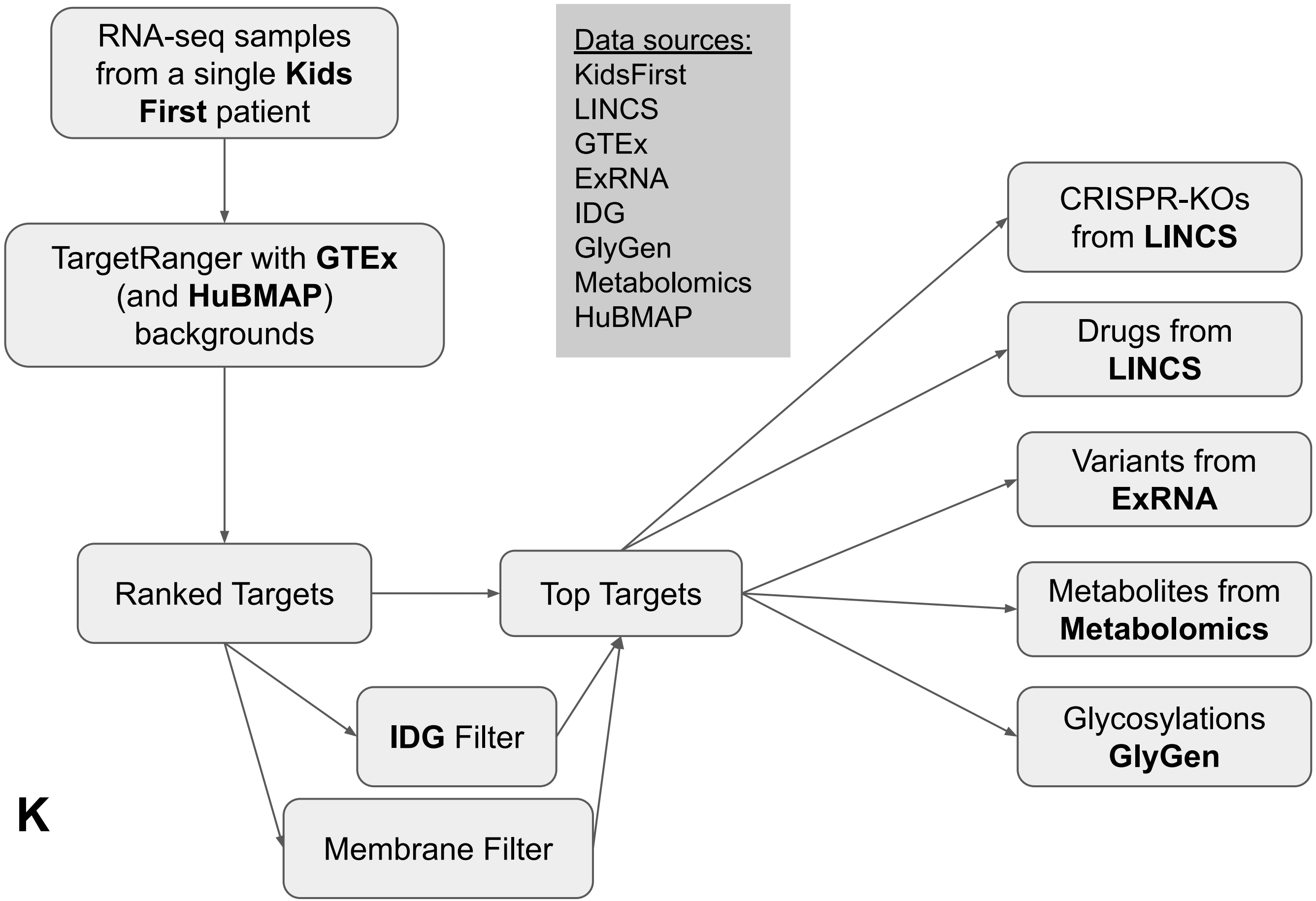

Fig. S1 K

Use Case 21 - Process a GSE Study and Perform Enrichment Analysis for the DEGs Against CF Gene Set Libraries

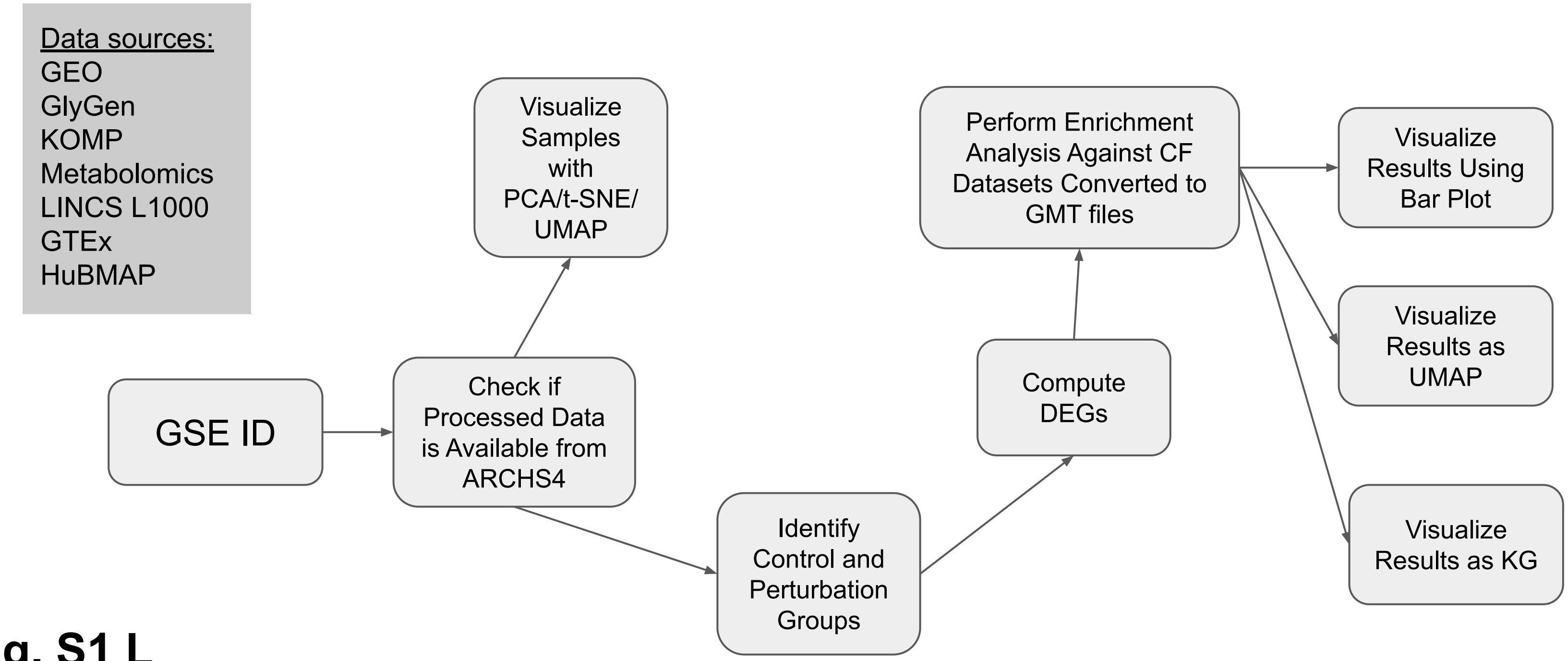

Fig. S1 L

Use Case 22 - Perform Kinase Enrichment Analysis Followed by Compound Identification from LINCS L1000 and Other Sources

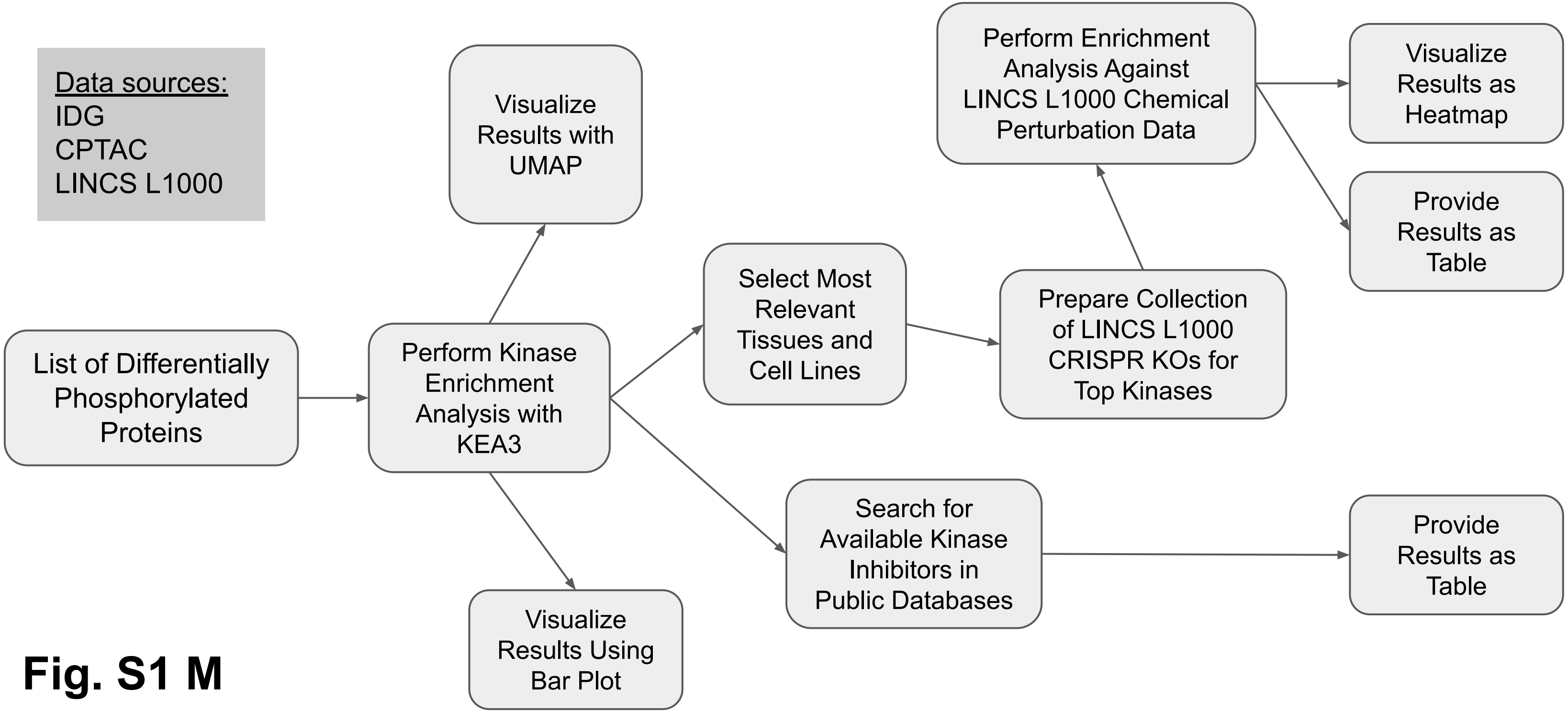

Fig. S1 M

Use Case 23 - Find Small Molecules and Drugs that Reverse Gene Expression in Young vs. Old Tissues from GTEx (LINCS)

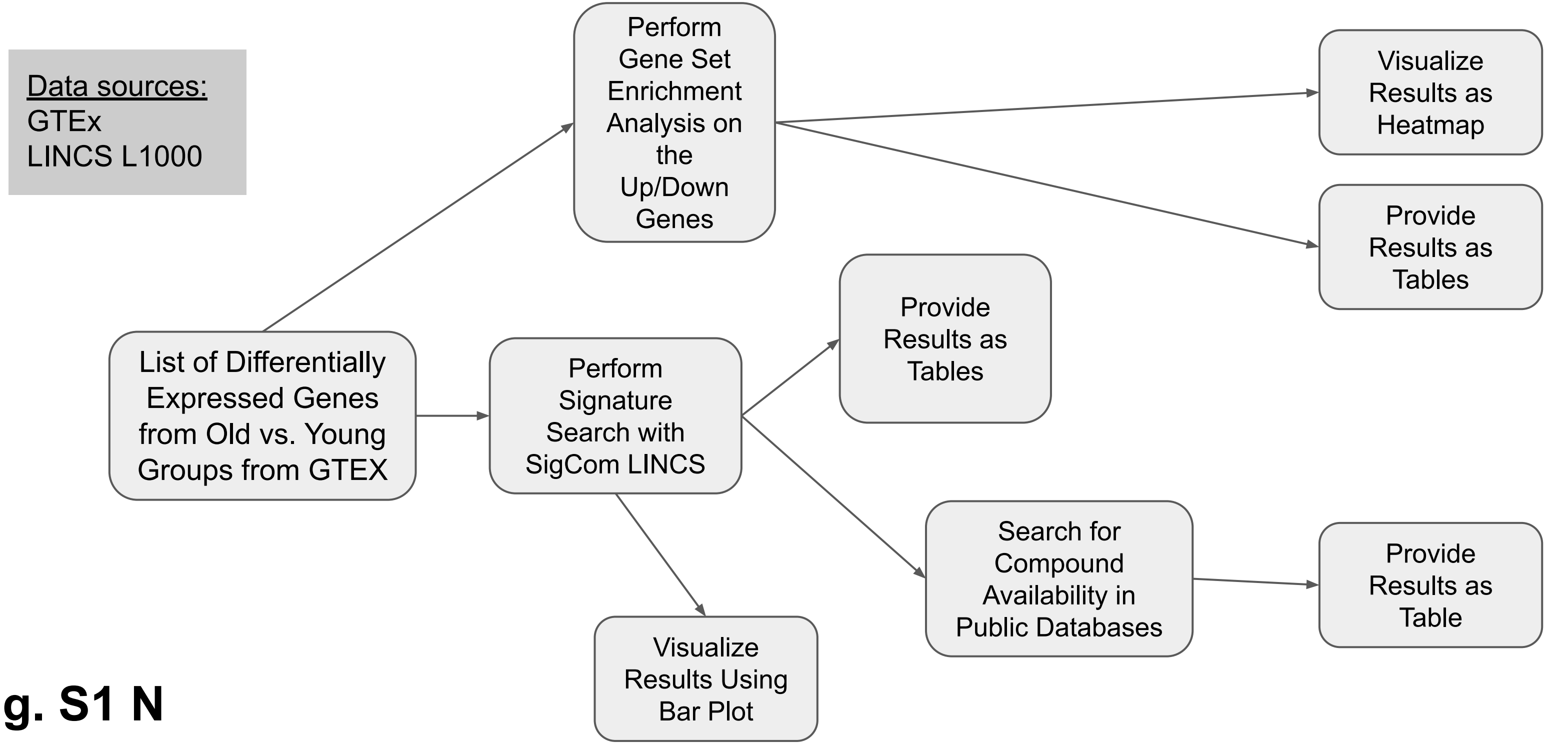

Fig. S1 N

Use Case 24 - Find Small Molecules and Drugs that Mimic Gene Expression Signature Changes Due to Exercise from MoTrPAC

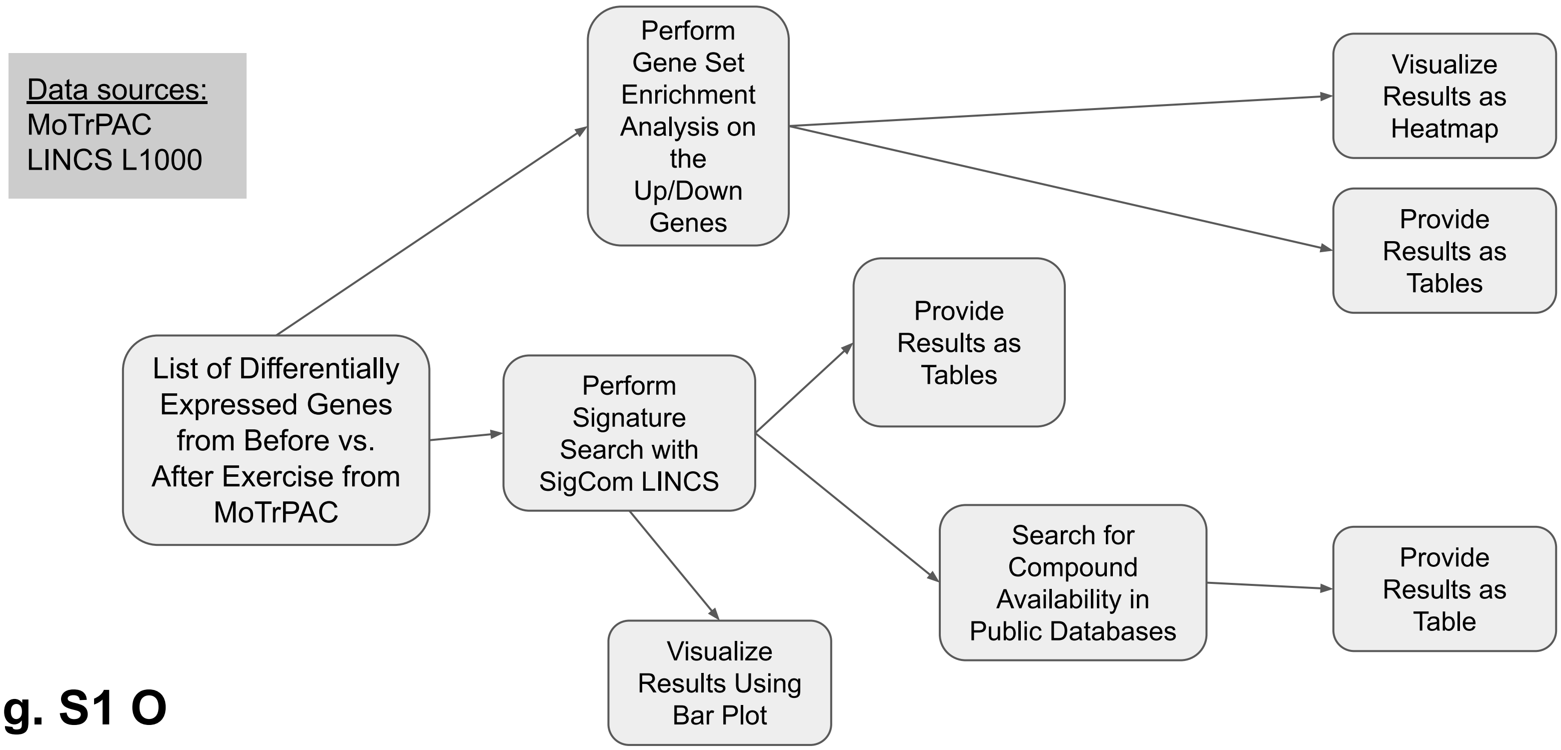

Fig. S1 O

Use Case 25 - Predict Small Molecules that are Likely to Induce Specific Birth Defects

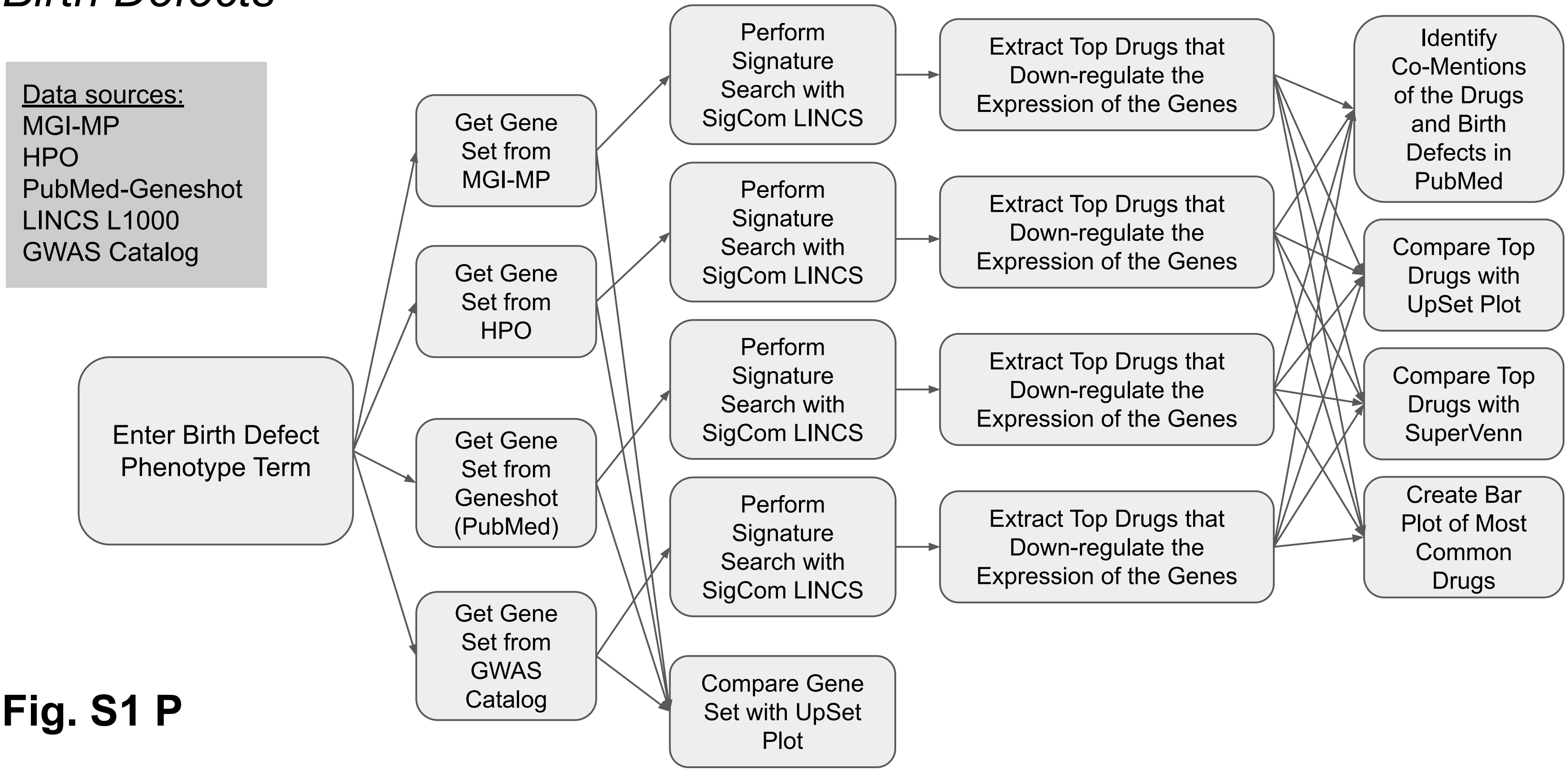

Fig. S1 P

Use Case 26 – PNPLA3 Related Proteins/Metabolites across DCCs

Data sources:
LINCS L1000
STRING
ChEA
GTEx
Enrichr
KEGG
GO
MSigDB
MW

Fig. S1 Q

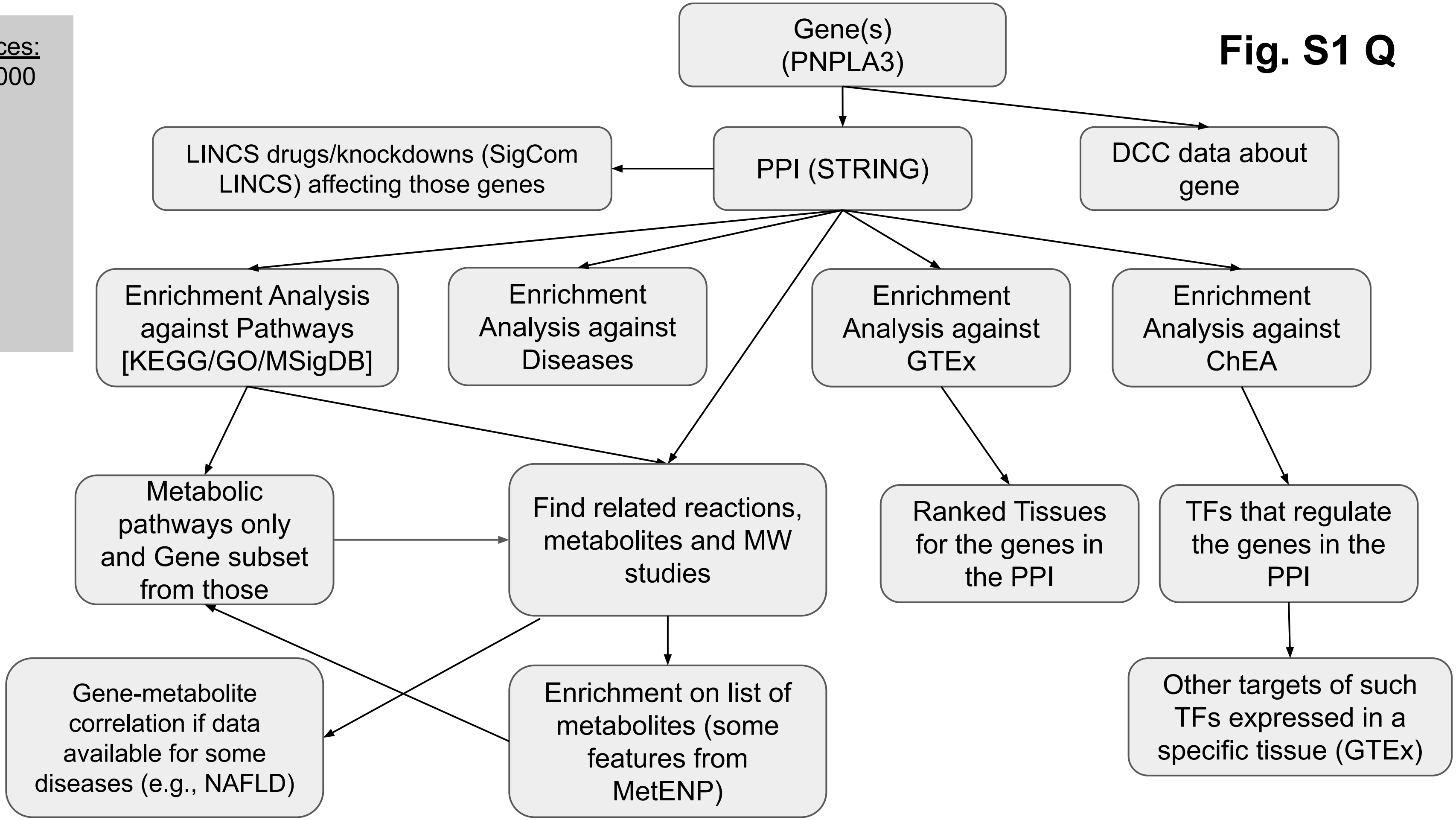

Fig. S1 R

Use Case 27 - Linking FAIR Regulatory Information for Genes, Regulatory Regions, and Variants

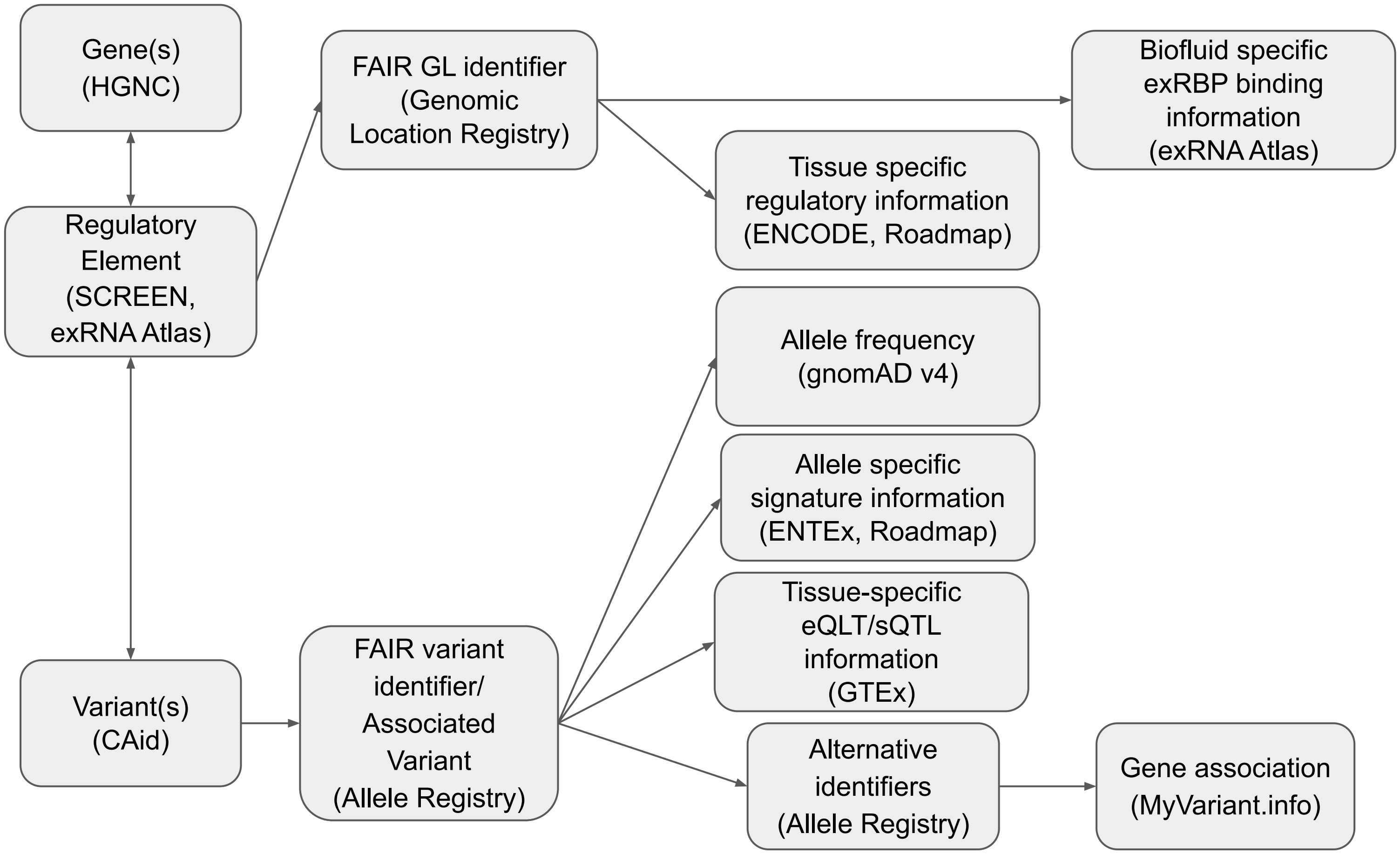

<u>Data sources:</u>
ClinGen Allele Registry
Kids First
ENCODE SCREEN
ENTEx
Roadmap
HuBMAP
GTEx
exRNA Atlas

Fig. S1 S

Use Case 28 - Discovery of De Novo Pathogenic Regulatory Variants from WGS Data

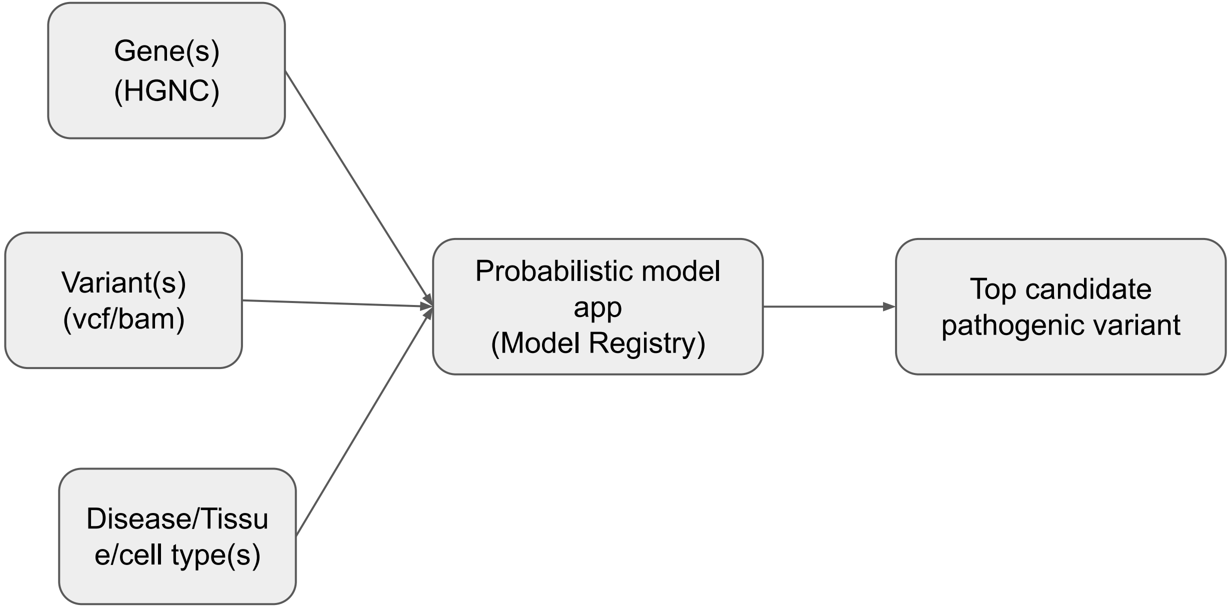

Data sources:
ClinGen Allele Registry
Kids First
ENCODE SCREEN
ENTEx
Roadmap
HuBMAP
GTEx
exRNA Atlas

Fig. S1 T

Use Case 29 - Form Novel Hypotheses with CFDE Gene Sets and Rummagene

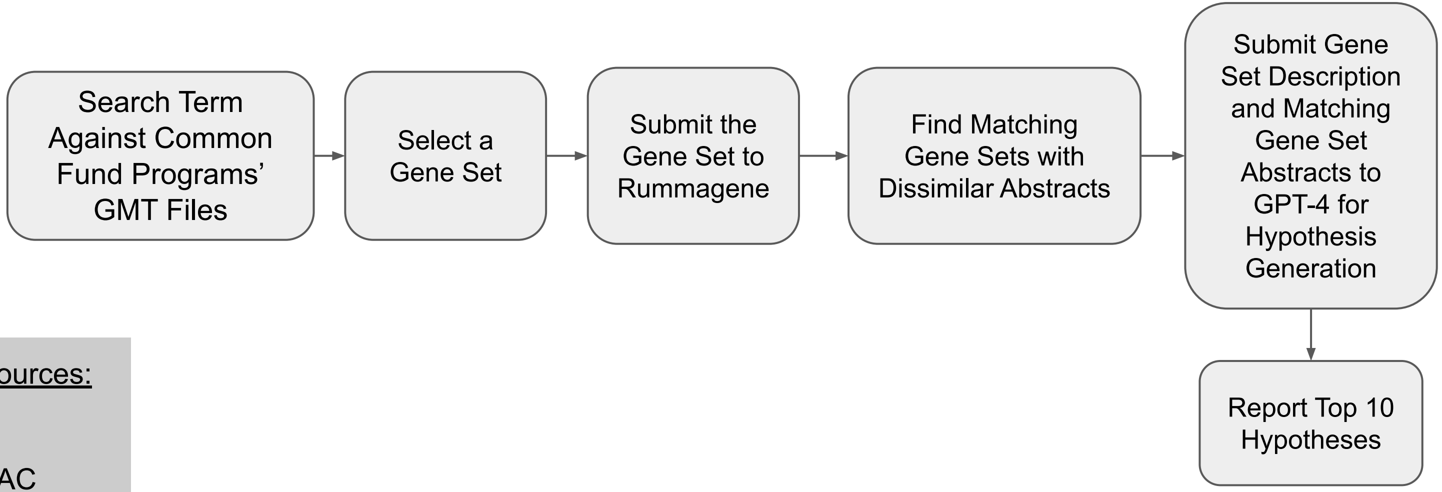

<u>Data sources:</u>
GTEx
LINCS
MoTrPAC
GlyGen
IMPC
Rummagene
MW
HuBMAP

Fig. S1 U

Use Case 31 - Find Small Molecule Mimickers for Single Gene Knockouts

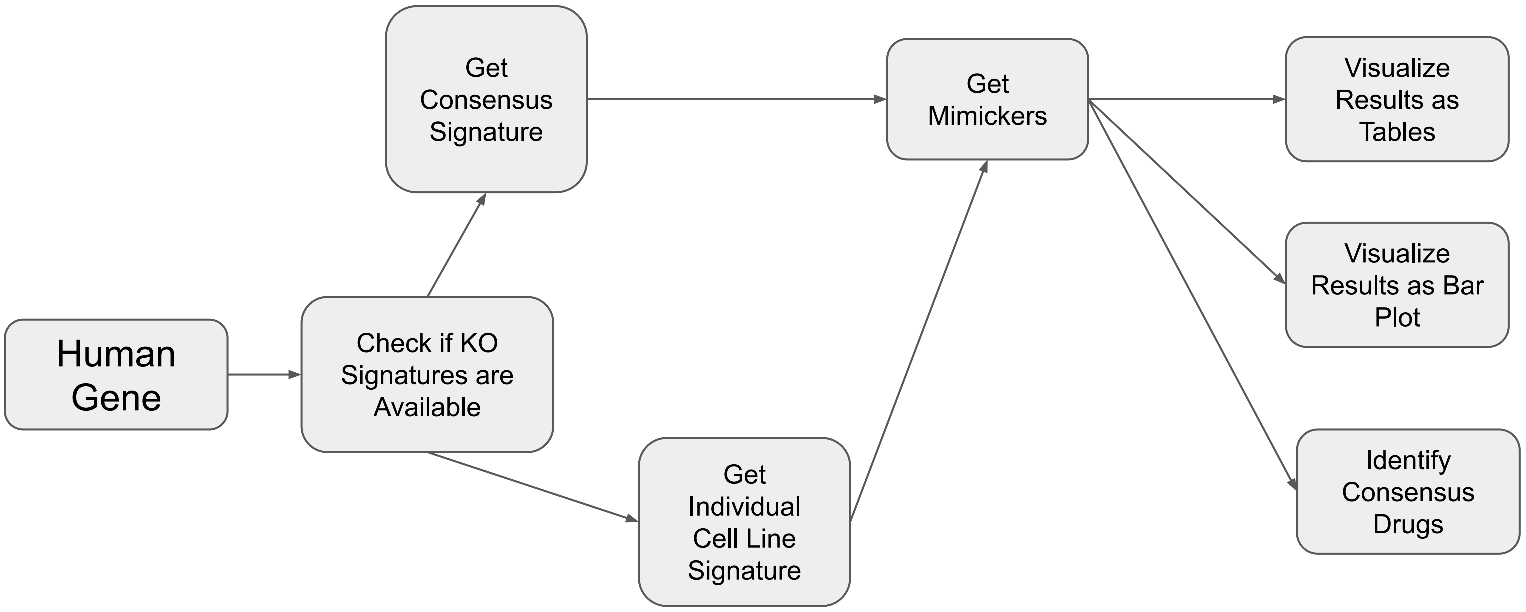

Data sources:
LINCS L1000

Fig. S1 V

Use Case 32 - Drug Identification for Pediatric Cancer Treatment in Clinical Settings

Data sources:
Kids First
LINCS
ChEMBL

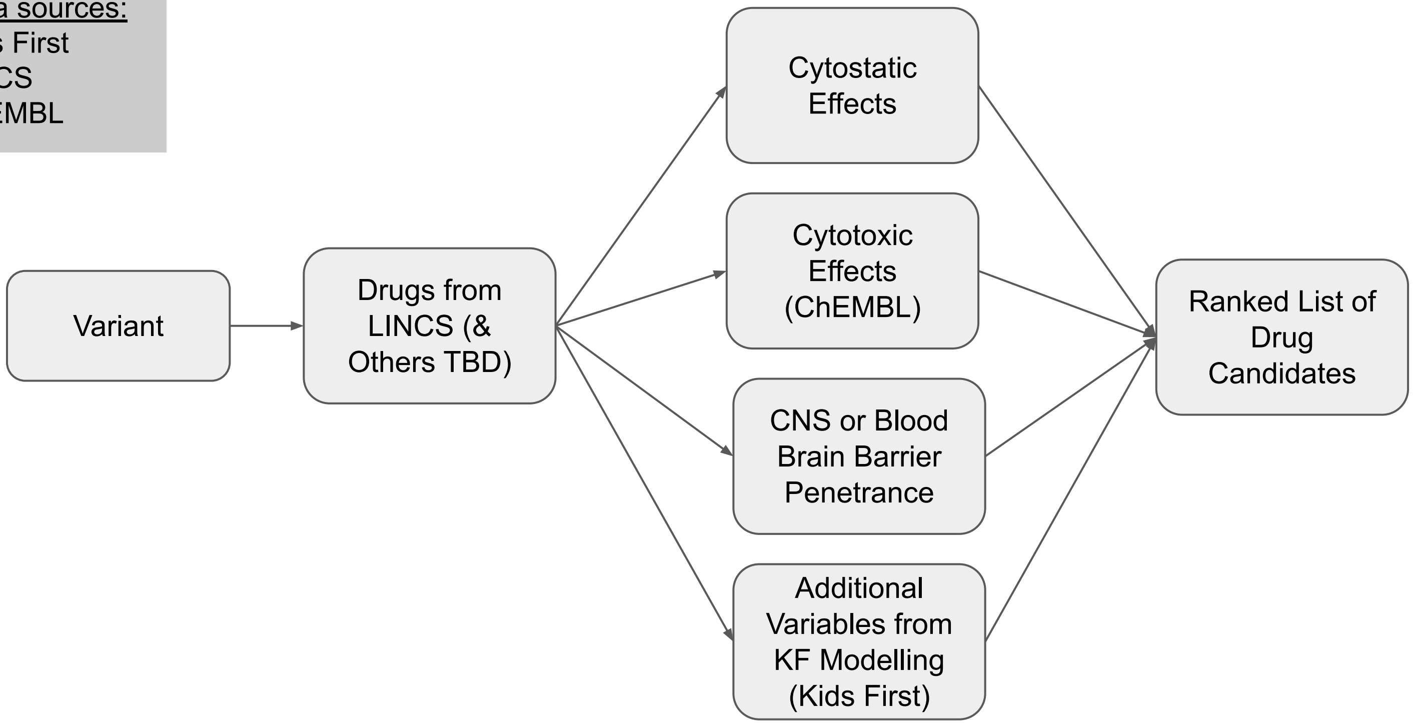

Supplement: S1 Fig — Before implementing metanodes to construct workflows, the workflows were sketched as flowchart diagrams. Note that not all nodes and links in the diagrams were implemented exactly how they were designed. (PDF) [file pcbi.1012901.s001.pdf]
