# Supplementary figures and images for: Playbook workflow builder: Interactive construction of bioinformatics workflows
Source: PLoS Comput Biol. 2025 Apr 3;21(4):e1012901. doi: 10.1371/journal.pcbi.1012901 (PMC11967941; doi:10.1371/journal.pcbi.1012901)

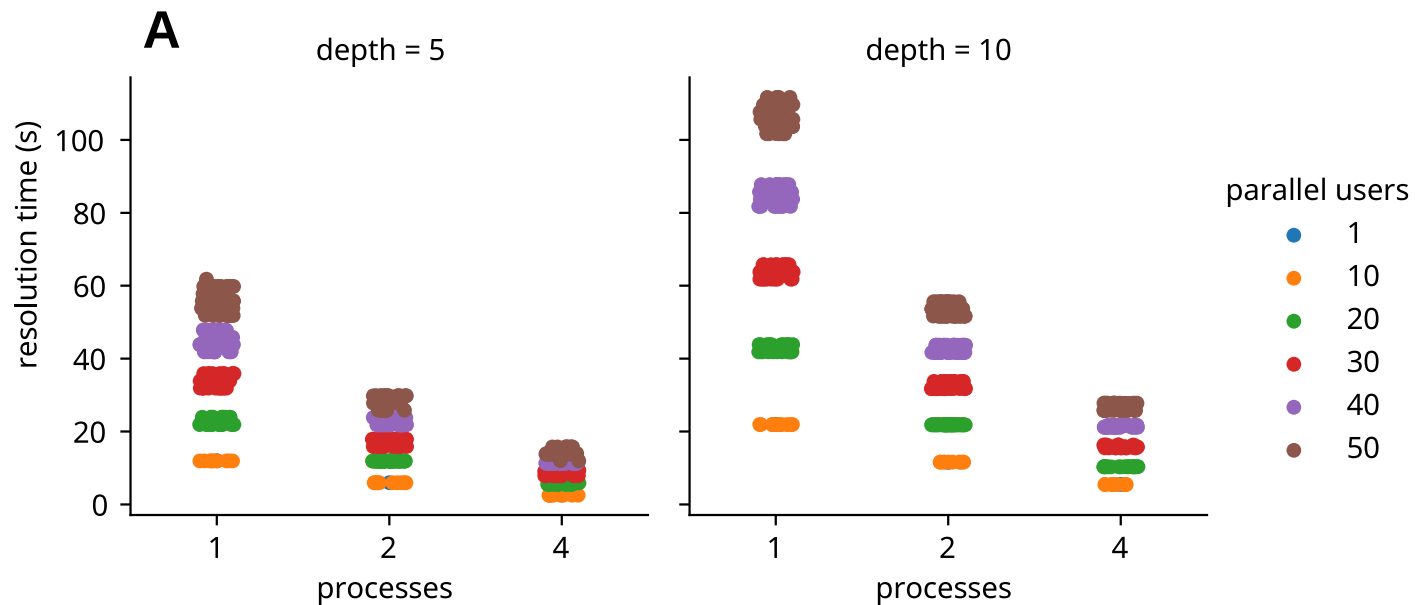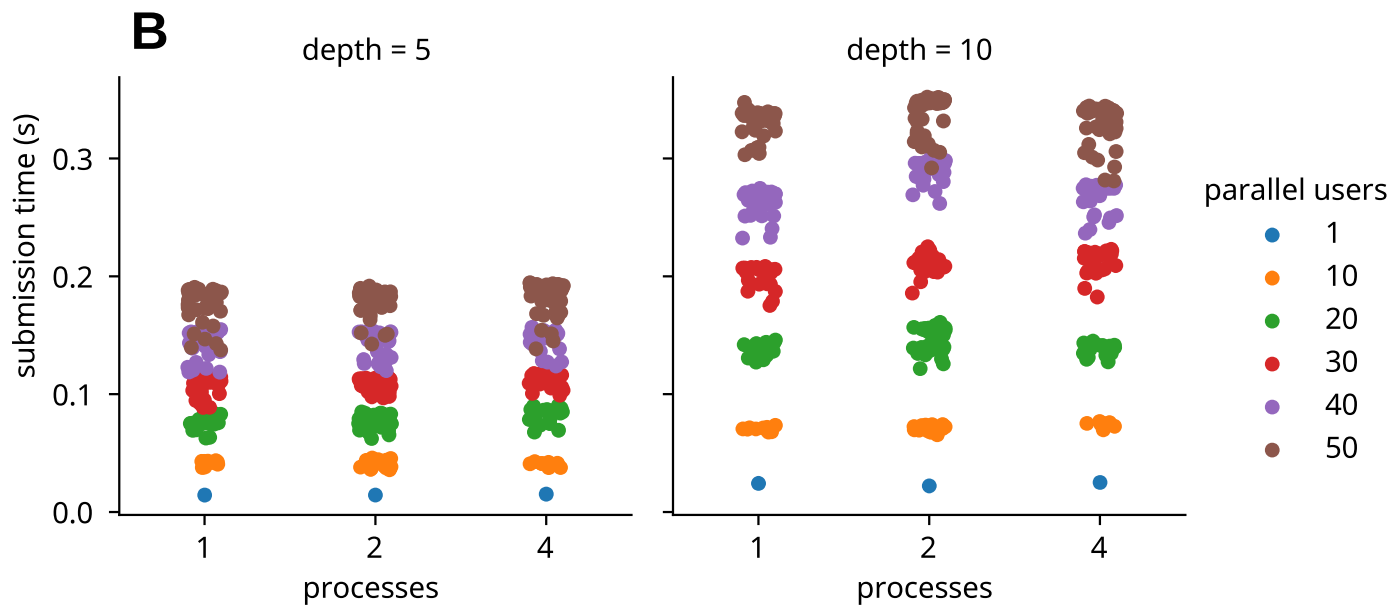

Supplement: S2 Fig — Users submitting workflows in parallel were simulated with the platform using between 1 and 50 parallel users submitting workflows of depth 5 and 10. Figure shows the time in seconds it took for the entire workflow to be completed for each individual simulated user (A), and the time it took to submit that workflow via the API (B). Simulations performed with 1, 2, and 4 horizontally scaled backend processes each with 5 worker threads. (PDF) [file pcbi.1012901.s002.pdf]
