# Supplementary material for: Playbook workflow builder: Interactive construction of bioinformatics workflows
Source: PLoS Comput Biol. 2025 Apr 3;21(4):e1012901. doi: 10.1371/journal.pcbi.1012901 (PMC11967941; doi:10.1371/journal.pcbi.1012901)
Supplement: Attachment — Submitted filename: Response-to-Reviewers.docx [file pcbi.1012901.s006.docx]

**Replies to the editors’ comments:**

**Editor:** Your manuscript is missing the following sections: Design and Implementation, and Availability and Future Directions. Please ensure that your article adheres to the standard Software article layout and order of Abstract, Introduction, Design and Implementation, Results, and Availability and Future Directions.

**Reply: Thank you for pointing this out. For the revised manuscript we added the missing sections as instructed.**

**Editor:** Please upload all main figures as separate Figure files in .tif or .eps format.

**Reply:** **We uploaded all figures as separate files in the specified format.**

**Editor:** Some material included in your submission may be copyrighted. According to PLOSu2019s copyright policy, authors who use figures or other material (e.g., graphics, clipart, maps) from another author or copyright holder must demonstrate or obtain permission to publish this material under the Creative Commons Attribution 4.0 International (CC BY 4.0) License used by PLOS journals. Please closely review the details of PLOSu2019s copyright requirements here: PLOS Licenses and Copyright. If you need to request permissions from a copyright holder, you may use PLOS's Copyright Content Permission form.

**Reply:** **We removed all materials from the manuscript that may require copyright permissions.**

**Editor:** Potential Copyright Issues:

i) Figures 1, 3B, 4, and 5. Please confirm whether you drew the images / clip-art within the figure panels by hand. If you did not draw the images, please provide (a) a link to the source of the images or icons and their license / terms of use; or (b) written permission from the copyright holder to publish the images or icons under our CC BY 4.0 license. Alternatively, you may replace the images with open source alternatives.

**Reply: We removed all icons and logos from external sources and replaced these with text and originally crafted display items. The SVG breadcrumb icons are either svg-traced logos that are significantly modified, manually constructed svg icons, or blueprintjs icons (apache 2.0), or material design icons (apache 2.0).**

**Editor:** ii) Figure 1 contains logos. We are not permitted to publish this under our CC BY 4.0 license,even with permission. We ask that you please remove or replace it.

**Reply: We removed all logos from figure 1 and replaced these with text boxes.**

**Editor:** iii) You stated in the legend of Figure 2 that "The permanent URL for accessing the workflow in the PWB platform is: [https://playbook-workflow-builder.cloud/report/d94b8b0a-81cc-708c-e200-e00ef3451da0."](https://urldefense.proofpoint.com/v2/url?u=https-3A__playbook-2Dworkflow-2Dbuilder.cloud_report_d94b8b0a-2D81cc-2D708c-2De200-2De00ef3451da0.-2522&d=DwQFaQ&c=shNJtf5dKgNcPZ6Yh64b-ALLUrcfR-4CCQkZVKC8w3o&r=ZqOe9FaWUIgM2PExbtMf60M1G00IDNwrruqJA9ldBZ8&m=fEnuW-aIho7u8plVGbZJNW2oJh_ZcPHc1kMLqGSP9WvRfi5_IQuBEvNBSmadZ0DC&s=9FyEYi0eHSgCfsH3w4zYCWWKrfIzy7rMrReFKm0fxPY&e=)

**Reply: We replaced this link with a DOI we created for the workflow using WorkflowHub:** [**https://doi.org/10.48546/WORKFLOWHUB.WORKFLOW.1237.2**](https://doi.org/10.48546/WORKFLOWHUB.WORKFLOW.1237.2)**.**

**Editor:** 4) Please amend your detailed Financial Disclosure statement. This is published with the article. It must therefore be completed in full sentences and contain the exact wording you wish to be published.

1) State the initials, alongside each funding source, of each author to receive each grant. For example: "This work was supported by the National Institutes of Health (####### to AM; ###### to CJ) and the National Science Foundation (###### to AM)."

2) State what role the funders took in the study. If the funders had no role in your study, please state: "The funders had no role in study design, data collection and analysis, decision to publish, or preparation of the manuscript.".

**Reply: We revised the Financial Disclosure statement as instructed.**

**Editor:** 5)Thank you for stating that “There are no primary data in the paper; the project’s source code with instructions is available from: [https://github.com/MaayanLab/Playbook-Workflow-Builder](https://track.editorialmanager.com/CL0/https:%2F%2Fgithub.com%2FMaayanLab%2FPlaybook-Workflow-Builder/1/010f01934e7a432d-eaa3dafe-018e-4a55-80c7-80d5f8ab2975-000000/nOB2bMdN_z12_8uRev2BKvB2WHje9NxLH1DR-nFcgTE=186).” We notice that there is a CC BY-NC-SA license on your data. We would encourage you to consider using a license that is no more restrictive than CC BY, in line with PLOS’ recommendation on licensing ([http://journals.plos.org/plosone/s/licenses-and-copyright)](http://track.editorialmanager.com/CL0/http:%2F%2Fjournals.plos.org%2Fplosone%2Fs%2Flicenses-and-copyright)/1/010f01934e7a432d-eaa3dafe-018e-4a55-80c7-80d5f8ab2975-000000/cydLfaZVHzdIpd4wLU2PfRNY7kOnpFhLWdNcMSs-ZGk=186).”

**Reply: We changed the license of the project to CC BY 4.0. We also deposited the code in Zenodo and provided the DOI near the link to GitHub.**

**Replies to the reviewers’ comments**

**Replies to Reviewer 1**

**Reviewer 1:** My main question is, given this would become entry 358 on
[https://s.apache.org/existing-workflow-systems](https://urldefense.proofpoint.com/v2/url?u=https-3A__s.apache.org_existing-2Dworkflow-2Dsystems&d=DwQFaQ&c=shNJtf5dKgNcPZ6Yh64b-ALLUrcfR-4CCQkZVKC8w3o&r=ZqOe9FaWUIgM2PExbtMf60M1G00IDNwrruqJA9ldBZ8&m=fEnuW-aIho7u8plVGbZJNW2oJh_ZcPHc1kMLqGSP9WvRfi5_IQuBEvNBSmadZ0DC&s=v2nFZ0r1mO5AqrSBuG5IpezDgzUld5D-DQQgQSRR3j4&e=) why we need another workflow system. The article hints at this, but does not make it clear. Galaxy is widely used and referenced by the article, along with perhaps more techie Snakemake and Nextflow; however the article shows little reflection on what makes Playbook different.

**Reply: Thank you for your comments. We’ve updated the text to elaborate on the justification of why we decided to create a completely new workflow platform instead of using or modifying existing platforms. The discussion about the unique features of the PWB platform is provided in the new section titled: *Reproducibility of workflows* and *Comparison to other Platforms*.**

**Reviewer 1:** The creation of metanodes for adding tools to Playbook seems similar to adding to Galaxy's toolshed, which currently have over 10.000 tools [https://toolshed.g2.bx.psu.edu/](https://urldefense.proofpoint.com/v2/url?u=https-3A__toolshed.g2.bx.psu.edu_&d=DwQFaQ&c=shNJtf5dKgNcPZ6Yh64b-ALLUrcfR-4CCQkZVKC8w3o&r=ZqOe9FaWUIgM2PExbtMf60M1G00IDNwrruqJA9ldBZ8&m=fEnuW-aIho7u8plVGbZJNW2oJh_ZcPHc1kMLqGSP9WvRfi5_IQuBEvNBSmadZ0DC&s=_s2US7khEEmDmy_3y9sSG9fT3GYRoTD4uU6Dkja1J9E&e=). The way of building-executing workflows step by step in Playbook is also clearly inspired by Galaxy, where a workflow can be retrospectively constructed from a data-driven history.

**Reply: We added some additional text to distinguish PWB metanodes from Galaxy toolshed tools. Specifically, we added the following text:**

**“In contrast with Galaxy [13], and the Galaxy ToolShed [108], PWB metanodes operate with semantic and runtime asserted JSON intermediaries instead of files. These artifacts make API usage more practical, and it enables custom rendering of information passed throughout the system. PWB components can also be implemented to receive user input, and components can have interactive visualizations. These features provide customization via user interaction. Examples of customized user interactions that are not possible with Galaxy, but present in PWB, include: autocomplete for user-input selection, extracting regions from an interactive set overlap visualization, interactive labeling of columns from a data table. Additionally, constructing a Galaxy workflow involves selecting from a massive list of all possible Galaxy supported tools and configuring each tool by providing it with all needed options and files. In contrast, PWB workflows start with a selection of a data file, or other information provided by the users. Once such selection is made, only the applicable tools for the next step are becoming available. This happens at each step of workflow construction. Each user step selection in the PWB environment yields a new workflow capable of executing, exporting, saving, and sharing with a persistent URL.”**

**Reviewer 1:** The authors claim workflow systems like Taverna were replaced by cloud-centric approaches. As former Taverna developer I would expand on this to mean a move from using public Web services to cloud/cluster based command line executions, which I will happily admit gave users greater scalability, stability and portability. Galaxy's tools for instance are primarily command line wrappers but some (particularly for data access) are REST API clients. The use of containers have also greatly improved the installation of such tools. But from this argument falls down as Playbook suggests using microservice architecture. On inspection of the source code I see that several metanodes are Node or Python-based, with dependencies installed into the main Playbook container, this is positive and could be made more clear in the manuscripts.

**Reply: Thank you for these comments. We added more details and clarity about this concept as follows:**

**“The PWB platform was designed to empower biomedical researchers without coding skills. Users can find and invoke bioinformatics workflows by leveraging the availability of public datasets, and commonly used data visualization methods. In contrast with other workflow engines, PWB users are provided with written details with citations about steps of the analysis. The PWB platform was initially created for a specific purpose of bringing to the surface datasets and tools developed in projects that received support from the NIH Common Fund. Due to this initial goal, many of the PWB components trigger external APIs and this feature of the platform has advantages and disadvantages. The use of federated microservices enables tool and repository owners to continuously improve and update these services independently of the PWB platform. Most repositories and web-based bioinformatics tools typically have the computational infrastructure to resolve queries in real-time, assembling all the data behind the service. Hence, it may become cost prohibitive for a single researcher, or a single platform to locally host these resources in one place. Additionally, many resources developed by Common Fund supported programs continue to evolve as additional data is gathered. The maintenance of these APIs and the data these APIs query pose a long-term sustainability challenge. While the PWB platform cannot guarantee the reproducibility of the results because federated knowledge continues to evolve, the PWB platform supports exporting reports. These exports contain the workflow, the output produced by every step in the workflow, and a timestamp of when the APIs were called. This description of the workflow can be interrogated, and the entire workflow can be imported into a Docker-served container using the same version of the original APIs.”**

**Reviewer 1:** It is unclear from the paper who is hosting and maintaining the microservices that Playbook relies on, and this should be a concern for reproducibility and longer term availability. In a sense the author's proposed approach is moving back to the Web Service model of Taverna, but with a central knowledge graph of consise types rather than looser typed BioCatalogue (now subsumed by [bio.tools](https://urldefense.proofpoint.com/v2/url?u=http-3A__bio.tools&d=DwQFaQ&c=shNJtf5dKgNcPZ6Yh64b-ALLUrcfR-4CCQkZVKC8w3o&r=ZqOe9FaWUIgM2PExbtMf60M1G00IDNwrruqJA9ldBZ8&m=fEnuW-aIho7u8plVGbZJNW2oJh_ZcPHc1kMLqGSP9WvRfi5_IQuBEvNBSmadZ0DC&s=m0xbe83XtAmGQj2eJVJrg4AXp20kiD8v3NqBrqYEzzk&e=) which use EDAM types).

If the Playbook system can itself dynamically deploy these microservices (the article does not say), similar to Galaxy's tools spinning up containers, then the longevity of Playbook workflows could look brighter.

**Reply:** **We recognize this caveat and added the following text to the Discussion section:**

**“It is important to note that PWB components are not inherently required to be external microservices. In fact, roughly half of the PWB components are not. These components operate without external APIs. Most PWB components that access large and evolving indexed databases, however, are wrappers around external APIs. The nature of evolving federated services makes assembling all parts of all workflows into an isolated environment is largely intractable. As the field rapidly evolves in data types and methods, out-of-date tools inevitably become less useful. Importantly, the PWB platform retains timestamped results so that reports persist, but re-execution of these reports is subject to change, providing the most recent knowledge from each microservice.”**

**Reviewer 1:**I would have liked to see more about how the Playbook type system was developed, and contrast it with earlier work of creating a single type-safe ecosystem of bioinformatics services, such as BioConductor, caGrid, BioMoby, SADI. Publications on Workflow Decay are particular relevant in this respect.

**Reply: Thank you for pointing out this gap in our discussion, we have noted these additional platforms in the Introduction and contrasted these services in the manuscript in a new section that compares PWB to other systems.**

**Reviewer 1:**The authors should be commended for implementing export to standards BCO, CWL, RO-Crate. This use of FAIR Computational Workflow serialisations can significantly help against longer term workflow decay and improve Reusability of Playbook workflows. However, if the CWL steps simply call public web microservices, these exports are just as vulnerable to service changes/downtime, and this should be admitted as a potential risk. They would also not benefit from cloud scalability on CWL sode. It is not clear which of the metadata annotations e.g. license are carried on to these formats, here again there is great potential.

**Reply: Thank you for your comments and suggestions. We have added text about the metadata annotations carried over to BCO, CWL, and RO-Crate standards which include: license, authorship information, versions, and methods. As noted, some steps in the PWB utilize external APIs, and we have since annotated these steps in an effort to ensure users are well aware of the caveats of using those services. We also added more text to the paper discussing this issue.**

**Reviewer 1:**It is not clear from article if Playbook workflows are or can be published in WorkflowHub, this is certainly achievable with RO-Crate export, but may require an equivalent import on Playbook side, e.g. embedding its native JSON definition of the workflow template,

The Workflow Run Crate profile has been adopted recently by Galaxy and CWL engines, and could be considered for more detailed provenance records beyond BCO and Workflow Crate features used by WorkflowHub, see our recent paper [https://doi.org/10.1371/journal.pone.0309210](https://urldefense.proofpoint.com/v2/url?u=https-3A__doi.org_10.1371_journal.pone.0309210&d=DwQFaQ&c=shNJtf5dKgNcPZ6Yh64b-ALLUrcfR-4CCQkZVKC8w3o&r=ZqOe9FaWUIgM2PExbtMf60M1G00IDNwrruqJA9ldBZ8&m=fEnuW-aIho7u8plVGbZJNW2oJh_ZcPHc1kMLqGSP9WvRfi5_IQuBEvNBSmadZ0DC&s=-b3N9syRK3kfqCnF7BQUTiNYnY4LN5yvX-0_4YDR-WI&e=)

**Reply:** **Thank you for this comment and suggestion. We have added the RO-Crate export feature to the PWB. We based our RO-Crate exports off of WorkflowHub’s representation of PWB exported CWL.**

**Reviewer 1:** Upon trying the Playbook, several very neat features seem to not be highlighted by the article:
- The user can document the workflow in a similar style to a Jupyter notebook, e.g. adding references
- Interactive visualisations e.g. LINCS, Enrichr
- Example values
- View In Graph navigation
- LLM integration not described much

**Reply: Thank you for pointing this out. For the revised manuscript we have added text to highlight these features.**

**Reviewer 1:** It was unclear to me if the Use Cases listed are meant to be reproducible, as I can't find distinction between a workflow template, a previous run, and my own rerun. Below are my observations:

- Use case 13: Input (example.h5ad?) is missing. Intermediate values are shown for the remaining steps, with visualisations. Final step Search PlyGen shows "Error: No matching protein product found."

**Reply: The missing example file was restored. The error message that no matching protein product was found is a legitimate error message in cases that there are no known GlyCans for a protein in the GlyGen database.**

- use case 1: : All example inputs are provided. Clicking "Recompute" on various steps do not seem to do anything.

**Reply: This was a bug that was fixed for the revised version.**

- Use case 11: Using the provided example "ACE2" works for the first steps, but fails in "Graph Plot". "MetGENE Metabolites with Gene Set" fails with `SyntaxError: Unexpected token.. DOCTYPE ... is not valid JSON". "MetGENE Studies Table" table is empty.

**Reply: These bugs were fixed for the revised version.**

- Use case 27: Does not seem to be public listed?

**Reply: Thank you for pointing this out. Use case 27 was renamed to use case 10 which was published, the text in the manuscript was updated accordingly.**

**Reviewer 1:** I was unable to find the RO-Crate export in the UI. This seems to be not yet merged into the main source code as the API endpoint does not work. The manuscript should be modified to reflect that RO-Crate export is experimental branch. From a cursory look, the RO-Crate is quite sensible and includes links to the native JSON, CWL and BCO outputs; however it is almost compatible WorkflowHub as the workflow files would be by URI references rather than embedded within a Zip file -- this output as a direct RO-Crate Metadata JSON from an API is what we now called Detached RO-Crate and will be part of RO-Crate 1.2

**Reply: Thank you for pointing this out. For the revised version of the manuscript and application we now fully support the RO-Crate feature and it has been merged and deployed.**

**Reviewer 1:** The source code of Playbook has been made available at but is not with an open source software license. Creative Commons License CC-BY-NC-SA 4.0 is NOT RECOMMENDED for software and NC is not open source as it restricts commercial use.

**Reply: We changed the license of the project to CC BY 4.0 and also deposited a snapshot of the code into Zenodo.**

**Reviewer 1:** Data availability statement should be modified to note that the software is not open source. Alternatively, a GNU license like GNU Affero General Public License may be appropriate if the authors want to prevent commercial use without open source any modifications. You should check the licenses of your Python and NodeJS dependencies for compatibility with your chosen license.

**Reply: We changed the license of the project to CC BY 4.0 and verified all installed Python and NodeJS dependencies are compatible with this license.**

**Reviewer 1:** I recommend enabling Zenodo archiving automatic from GitHub release -- this would give a code citation DOI persistent identifier authors can cite from the manuscript (in case GitHub goes).

**Reply: Thank you for this suggestion. We enabled Zendo archiving for PWB releases.**

**Reviewer 1:** Overall I welcome this contribution, but would primarily ask the authors to point out contrasts to other systems for potential consumers. Table S.1 is very incomplete and is more of an illustration, the paper should rather reference review papers and guides like [https://doi.org/10.1016/j.future.2017.01.012](https://urldefense.proofpoint.com/v2/url?u=https-3A__doi.org_10.1016_j.future.2017.01.012&d=DwQFaQ&c=shNJtf5dKgNcPZ6Yh64b-ALLUrcfR-4CCQkZVKC8w3o&r=ZqOe9FaWUIgM2PExbtMf60M1G00IDNwrruqJA9ldBZ8&m=fEnuW-aIho7u8plVGbZJNW2oJh_ZcPHc1kMLqGSP9WvRfi5_IQuBEvNBSmadZ0DC&s=XuI9vdeh7olOtCBp7QqlwBHCYKcAY-dk58PSpTEF_40&e=) [https://doi.org/10.1016/j.patter.2021.100322](https://urldefense.proofpoint.com/v2/url?u=https-3A__doi.org_10.1016_j.patter.2021.100322&d=DwQFaQ&c=shNJtf5dKgNcPZ6Yh64b-ALLUrcfR-4CCQkZVKC8w3o&r=ZqOe9FaWUIgM2PExbtMf60M1G00IDNwrruqJA9ldBZ8&m=fEnuW-aIho7u8plVGbZJNW2oJh_ZcPHc1kMLqGSP9WvRfi5_IQuBEvNBSmadZ0DC&s=s1-dRv8KsBDqts2HmG8PwChqqu-T8qWBna5h0K70tF0&e=) [https://doi.org/10.1038/s41592-021-01254-9](https://urldefense.proofpoint.com/v2/url?u=https-3A__doi.org_10.1038_s41592-2D021-2D01254-2D9&d=DwQFaQ&c=shNJtf5dKgNcPZ6Yh64b-ALLUrcfR-4CCQkZVKC8w3o&r=ZqOe9FaWUIgM2PExbtMf60M1G00IDNwrruqJA9ldBZ8&m=fEnuW-aIho7u8plVGbZJNW2oJh_ZcPHc1kMLqGSP9WvRfi5_IQuBEvNBSmadZ0DC&s=fBMf6ALxAZKBAyZwlVzfMR0_NQB5ME7-ZXOJm2hWKd0&e=) [https://doi.org/10.1007/978-1-4939-9074-0_24](https://urldefense.proofpoint.com/v2/url?u=https-3A__doi.org_10.1007_978-2D1-2D4939-2D9074-2D0-5F24&d=DwQFaQ&c=shNJtf5dKgNcPZ6Yh64b-ALLUrcfR-4CCQkZVKC8w3o&r=ZqOe9FaWUIgM2PExbtMf60M1G00IDNwrruqJA9ldBZ8&m=fEnuW-aIho7u8plVGbZJNW2oJh_ZcPHc1kMLqGSP9WvRfi5_IQuBEvNBSmadZ0DC&s=wgT93CYSoBjuC-4KG-hzva8ZQbqliQURb6PY11sVHrs&e=) [https://doi.org/10.1371/journal.pcbi.1008622](https://urldefense.proofpoint.com/v2/url?u=https-3A__doi.org_10.1371_journal.pcbi.1008622&d=DwQFaQ&c=shNJtf5dKgNcPZ6Yh64b-ALLUrcfR-4CCQkZVKC8w3o&r=ZqOe9FaWUIgM2PExbtMf60M1G00IDNwrruqJA9ldBZ8&m=fEnuW-aIho7u8plVGbZJNW2oJh_ZcPHc1kMLqGSP9WvRfi5_IQuBEvNBSmadZ0DC&s=1eU0aQ4ah1etGXEG96NIZP_trupCwDw9at4eZpAn6P4&e=)

**Reply: Thank you for these additional references. We have included them as references and updated Table S1 accordingly.**

**Reviewer 1:** I also have reservations with regards to reproducibility/rerunnability for a microservice-based environment, as I was unable to reproduce the listed use cases fully. A section should be added to discuss this.

**Reply: We appreciate this concern. Although we have since fixed several issues related to the published use cases, and also made sure to save time stamped outputs on saved workflows, we agree that more discussion about reproducibility is merited. The time stamping of workflows ensures that older workflows can be viewed even if in the future services change on how they operate and the data they return. We added a new section to the discussion that enlists the caveats of relying on external API-based workflow steps. We also discuss what we have done to mitigate some of those caveats.**

**Reviewer 1:** The RO-Crate support should either be merged or described as experimental, so that the live website matches the manuscript. (I recommend merge and name it Detached RO-Crate).

**Reply: Thank you for pointing this out, the RO-Crate support has been merged.**

**Reviewer 1:** Most of the citations are missing DOIs, ensure these are included in the print version.

**Reply: We used the Paperpile’s PLOS Computational Biology citation style. We will ensure that all the DOIs are at a later stage if required.**

Citation 15 for CWL specification should be replaced with journal paper

Citation 9-11 for Galaxy should include newest publication

**Reply: We replaced the citation to CWL (10.1145/3486897), and Galaxy (10.1093/nar/gkae410) as suggested. This is much appreciated.**

**Reviewer 1:**

p7. "although mostly underdeveloped, bioinformatics workflow engines... already exists"
This claim is unfounded; if anything, the most mature workflow engines are developed for bioinformatics.
Remove "Mostly underdeveloped".

p7. Clarify that Table S1 is not an extensive list but a sample.
S1 includes Taverna, but is missing X for "Graphical interface for workflow construction" and "Pre-defined modules". See

S1 is using PubMed ID instead of DOI for citations, meaning that citations are missing for CWL , WDL , WorkflowHub , taverna

p7. "... Galaxy..Snakemake..Nextflow..... These newer platforms rely on ... The two leading standards for workflow languages.. CWL..WDL"
This seem to apply that Galaxy, Snakemake and Nexflow support CWL and WDL, however, only Galaxy has (experimental) support for CWL. Rephrase, e.g. "In addition, two leading standards.."

p7. "Other examples of community standards ... BioCompute... and WorkflowHub"
WorkflowHub is not a community standard, but a registry. The community standard used by WorkflowHub is (as the next sentence explains), RO-Crate.
In particular, WorkflowHub uses a profile of RO-Crate that is called Workflow RO-Crate [https://w3id.org/workflowhub/workflow-ro-crate/1.0](https://urldefense.proofpoint.com/v2/url?u=https-3A__w3id.org_workflowhub_workflow-2Dro-2Dcrate_1.0&d=DwQFaQ&c=shNJtf5dKgNcPZ6Yh64b-ALLUrcfR-4CCQkZVKC8w3o&r=ZqOe9FaWUIgM2PExbtMf60M1G00IDNwrruqJA9ldBZ8&m=fEnuW-aIho7u8plVGbZJNW2oJh_ZcPHc1kMLqGSP9WvRfi5_IQuBEvNBSmadZ0DC&s=nxzpFFOIG1F7lSgIzKF9Tm1hL4Von7vdndHJ45HLvYw&e=)

p7. Federated Knowledge Graphs: Considerations of former work could mention BioConductor , BioMoby , SADI , caBIG -- these are all at least a decade old.

p8. add citations or urls for CF and CFDE.

p9. persistent urls are not exposed in the user interface as such. A persistent url should use a PID provider, e.g. DataCite/DOI, [https://w3id.org/](https://urldefense.proofpoint.com/v2/url?u=https-3A__w3id.org_&d=DwQFaQ&c=shNJtf5dKgNcPZ6Yh64b-ALLUrcfR-4CCQkZVKC8w3o&r=ZqOe9FaWUIgM2PExbtMf60M1G00IDNwrruqJA9ldBZ8&m=fEnuW-aIho7u8plVGbZJNW2oJh_ZcPHc1kMLqGSP9WvRfi5_IQuBEvNBSmadZ0DC&s=AC5YyaNWRkpYtgbbx-XgrNoI1wubfWwVgN7Trnt1XDM&e=) or [https://purl.archive.org/](https://urldefense.proofpoint.com/v2/url?u=https-3A__purl.archive.org_&d=DwQFaQ&c=shNJtf5dKgNcPZ6Yh64b-ALLUrcfR-4CCQkZVKC8w3o&r=ZqOe9FaWUIgM2PExbtMf60M1G00IDNwrruqJA9ldBZ8&m=fEnuW-aIho7u8plVGbZJNW2oJh_ZcPHc1kMLqGSP9WvRfi5_IQuBEvNBSmadZ0DC&s=rnCLFxUZK0r1q8h1HvlQ93kIfaSGbFPCj5d0OYaVbDs&e=) -- urls shown in the interface are called "temporary links", indicating they are _not_ persistent?

p13 - p15. Please add the persistent urls for each use case (see above)

p18. "operated-on at runtime in a type-safe manner through runtime-based type checkin" -- avoid repetition of "type" and "runtime"

**Reply: Thank you for making these suggestions to edit the text. We have applied all the suggested changes. We also updated table S1 with DOIs. We have also updated the text to clarify that the term persistence is used to refer to the fact that every stage of workflow construction is saved, and we are aware that this is not a guarantee of URL persistence. Nonetheless, we created persistent URLs for the published use cases with Workflowhub.**

**Replies to Reviewer 2**

**Reviewer #2:** This paper introduces a meaningful contribution to bioinformatics by addressing current challenges in workflow integration, usability, and reproducibility. PWB leverages a comprehensive ecosystem of APIs and a novel KRG framework, offering a scalable solution to handle diverse bioinformatics data types. The inclusion of 561 metanodes provides flexibility, and the capacity to visualize results and reuse workflows enhances the utility of the platform for both novice and experienced users. This paper includes strengths in terms of the innovation in workflow construction, standards compliance, and real-world application. The platform’s use of a chatbot powered by a large language model (LLM) for workflow guidance stands out as an innovative approach to reduce technical barriers, making the platform accessible. By aligning with CWL and BCO standards, PWB supports workflow portability and interoperability, which are crucial for collaborative and reproducible research. The detailed use cases demonstrate PWB's potential in handling real-world bioinformatics challenges, showcasing its adaptability across different biomedical research domains.

**Reply: Thank you for recognizing the value of the platform.**

**Reviewer #2:** Performance evaluation using empirical benchmarks could make this paper persuasive. Can you compare PWB's efficiency and accuracy with other workflow tools, such as Galaxy or Snakemake?

**Reply: We have added more text to further distinguish the scope of the PWB as a graphical domain-specific language (DSL) and workflow manager as opposed to a workflow execution system such as Galaxy and Snakemake. We added the following to the discussion section:**

**“Overall, the PWB platform is independent of the workflow execution system. This means that an existing workflow tool, like snakemake or CWL runner, could be employed with no change to the user experience. Currently, unresolved executions are queued in a postgreSQL database, and a horizontally scalable set of workers execute the tasks as they come in, while skipping tasks with unresolved dependencies. The PWB platform's primary focus is guiding the user through the analysis steps where provenance is fully captured. This produces a reusable workflow. Users can also “bring their own” workflow execution service via the CAVATICA integration. This feature could be expanded to support any GA4GH compatible workflow execution service, mostly eliminating the need for users to depend on the pooled resources that we can provide for the community.”**

**Reviewer #2:**  While the platform's scalability is theoretically addressed, more details on its performance under high data loads or with complex workflows would be beneficial.

**Reply: We have tested the PWB’s ability to support a high number of users and demonstrated that the system is horizontal scalability. Users with demanding jobs should also use the CAVATICA integration feature. We added the following to the discussion section about this issue:**

**“For computationally demanding jobs, and for working with patient protected private data, users of the PWB should execute their workflows via the CAVATICA integration. In CAVATICA, users can run any computationally intensive workflow with any GA4GH compatible workflow execution service. The CAVATICA integration can be used to reduce the burden of utilization of PWB native pooled resources available for the community of most PWB users.”**

**Reviewer #2:**  User studies or feedback on the interface and workflow creation experience would help substantiate the claim that PWB is user-friendly for non-experts.

**Reply: Thank you for this comment. This reminded us that we forgot to mention in the manuscript that we already conducted three user events and received ample feedback from users. We added this text to the revised manuscript:**

**“So far, we have conducted three use-a-thon events that introduced the PWB to new users. These users provided valuable feedback about the platform that we fully implemented. The users in these three events were both “regular” users that worked with the PWB via the UI, and “power” users that contributed new PWB components. The “regular” users provided feedback on how to improve the UI, while the “power” users provided feedback on the documentation and SDK for contributing PWB components.”**
